# Deletion of the *Mycobacterium tuberculosis cyp138* gene leads to changes in membrane-related lipid composition and antibiotic susceptibility

**DOI:** 10.3389/fmicb.2024.1301204

**Published:** 2024-03-25

**Authors:** Yun Lu, Hongtong Chen, Zhiyuan Shao, Lang Sun, Congran Li, Yu Lu, Xuefu You, Xinyi Yang

**Affiliations:** ^1^Beijing Key Laboratory of Antimicrobial Agents, Institute of Medicinal Biotechnology, Chinese Academy of Medical Sciences and Peking Union Medical College, Beijing, China; ^2^Division for Medicinal Microorganisms-related Strains, CAMS Collection Center of Pathogenic Microorganisms, Beijing, China; ^3^State Key Laboratory of Bioactive Substances and Functions of Natural Medicines, Institute of Medicinal Biotechnology, Chinese Academy of Medical Sciences & Peking Union Medical College, Beijing, China; ^4^Beijing Key Laboratory of Drug Resistance Tuberculosis Research, Beijing Tuberculosis and Thoracic Tumor Research Institute, and Beijing Chest Hospital, Capital Medical University, Beijing, China

**Keywords:** *Mycobacterium tuberculosis*, CYP138, gene knock-out, membrane-related lipids, antibiotics susceptibility, triacylglycerol

## Abstract

**Introduction:**

*Mycobacterium tuberculosis* (*Mtb*), the main cause of tuberculosis (TB), has brought a great burden to the world's public health. With the widespread use of *Mtb* drug-resistant strains, the pressure on anti-TB treatment is increasing. Anti-TB drugs with novel structures and targets are urgently needed. Previous studies have revealed a series of CYPs with important roles in the survival and metabolism of *Mtb*. However, there is little research on the structure and function of CYP138.

**Methods:**

In our study, to discover the function and targetability of CYP138, a *cyp138*-knockout strain was built, and the function of CYP138 was speculated by the comparison between *cyp138*-knockout and wild-type strains through growth curves, growth status under different carbon sources, infection curves, SEM, MIC tests, quantitative proteomics, and lipidomics.

**Results and discussion:**

The knockout of *cyp138* was proven to affect the *Mtb*'s macrophage infection, antibiotics susceptibility, and the levels of fatty acid metabolism, membrane-related proteins, and lipids such as triacylglycerol. We proposed that CYP138 plays an important role in the synthesis and decomposition of lipids related to the cell membrane structure as a new potential anti-tuberculosis drug target.

## 1 Introduction

Tuberculosis (TB), caused mainly by *Mycobacterium tuberculosis* (*Mtb*), is the leading cause of death from a single infection. WHO reported that an estimated 10.6 million people (95% UI: 9.9–11.4 million) fell ill with TB in 2022 worldwide, and China was one of the highest TB burden countries (World Health Organization, 2023). With the continuous and widespread use of first-line anti-tuberculosis drugs, multidrug-resistant (MDR) and extensively drug-resistant (XDR) TB appeared and posed great threats to public health (Mabhula and Singh, 2019). Anti-TB drugs with novel structures and targets are urgently needed to prevent the prevalence of drug-resistant strains.

Cytochromes P450s (CYPs) are monooxygenases that usually interact with flavoprotein and/or iron-sulfur central redox partners to perform catalysis (Lu et al., 2017). They are found in diverse species, from bacteria to vertebrates, and are known to be involved in the primary and secondary metabolisms of organisms (Nelson, 2018). CYPs from humans are closely related to the metabolism of numerous exogenous (i.e., drugs and polycyclic aromatic hydrocarbons) and endogenous molecules (i.e., steroids, vitamins, eicosanoids, and neurotransmitters), while CYPs from bacteria were found to be involved in species-specific biological processes such as antibiotic synthesis and nitric oxide reduction (Munro et al., 2003; McLean et al., 2010; Elfaki et al., 2018; Msomi et al., 2021).

The determination of the *Mtb* H37Rv genome sequence has revealed that 20 different CYP genes exist, indicating their vital roles in the survival or pathogenicity of *Mtb* H37Rv (McLean et al., 2006; Hudson et al., 2012). For the past few decades, *Mtb* CYPs have attracted lots of attention from researchers, and some of them have been structurally and functionally characterized. CYP51B1 (the first revealed prokaryotic sterol demethylase in prokaryotes) has been proven to be a promising anti-TB drug target (Podust et al., 2001). CYP124, CYP125, and CYP142 were found to be related to cholesterol metabolism (Johnston et al., 2012). CYP121 is shown to be essential for the viability of *Mtb*, and screening has been performed to find inhibitors (Kavanagh et al., 2016). CYP130 has been identified as an individually expressed protein, along with CYP51 and CYP121 (Ouellet et al., 2008). CYP128 is sufficient for the biosynthesis of sulfomenaquinone from menaquinone, which is related to *Mtb* virulence (Sogi et al., 2016). CYP141 is an intermediary metabolic and respiratory protein, whose gene was proved to be a good genotypic marker for direct detection of *Mtb* (Darban-Sarokhalil et al., 2011). Furthermore, CYP126, CYP143, and CYP144 were also purified and crystallized (Swami, 2015; Chenge et al., 2016). CYP139 was predicted to play a role in the synthesis of secondary metabolites in mycobacterial species (Syed et al., 2019).

CYP138 belongs to the *Mtb* CYPs, whose structures and functions are still unclear. Although transposon insertion mutagenesis (TRaSH)-based gene-deficient screening tentatively suggested that *cyp138* is non-essential for the survival of *Mtb* (Murphy and Brown, 2007), phylogenetic tree analysis of the amino acid sequences of CYP138 from the NCBI protein database revealed it highly conserved (99%) across *Mtb* complex species, including *Mycobacterium canettii, Mtb* variant bovis BCG, etc. (Supplementary Figure S1), suggesting the possibility of species-specific functions that we do not yet know. In addition, in a study on the influence of HIV on the evolution of *Mtb*, it was vaguely mentioned that CYP138 may indirectly interact with human proteins, thereby functionally interacting with HIV proteins (Koch et al., 2017). However, to date, little is known about the more detailed roles of the CYP enzyme.

To better understand the function and roles of CYP138 in *Mtb*, in our study, *cyp138* was knocked out from the *Mtb* genome by homologous recombination, and differences in the surface morphology, growth curve, growth status under different carbon sources, ability to infect macrophages, and antimicrobial susceptibility among *Mtb* H37Rv (wild-type), *cyp138-*knockout (Δ138), and *cyp138* complementary strains (Δ138-C) were evaluated. Proteomics and lipidomics were helpful tools for investigating mechanisms and elucidating the functions and roles of targeted proteins or genes (Jain et al., 2007; Qin et al., 2023). A more diverse dataset of molecular changes can be acquired by a multi-omics approach, which can help us with the functional clarification of targeted proteins or genes. Thus, to further characterize the specific effects or consequences of *cyp138*-knockout on profiles of protein expression and lipid production in *Mtb*, quantitative proteomes and lipidomes were used for large-scale comparative analysis of protein and lipid metabolite changes in the *cyp138-*knockout strain, and targeted proteomes based on parallel reaction monitoring (PRM) and thin layer chromatography (TLC) were used to confirm the changes.

## 2 Materials and methods

### 2.1 Bacterial strains and growth conditions

The wild-type and Δ138 strains were grown in the 7H9 medium supplemented with 0.2% (v/v) glycerol, 0.05% (v/v) Tween 80, and 10% (v/v) oleic acid albumin dextrose catalase [OADC, Becton, Dickinson, and Company (BD), United States] at 37°C. The Δ138-C was cultured in the 7H9 broth mentioned above, supplemented with an additional 10 μg·ml^−1^ (Gentamicin, GM). All strains were cultured on the 7H10 culture plate supplemented with 10% OADC at 37°C for 14 days.

### 2.2 Vector construction

**Figure 1:**
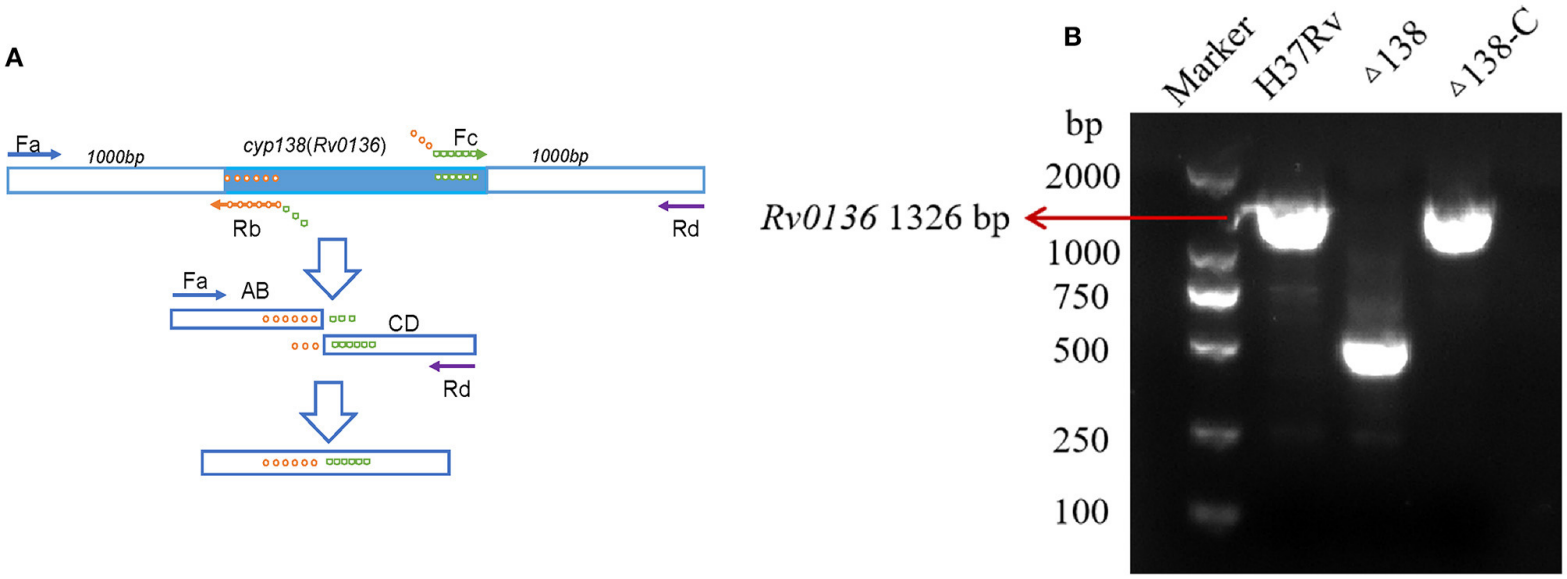
**(A)** Gene deletion by overlap extension PCR. For the first step, four primers were needed; the products of the first step were used for the template of the second step. **(B)** Primers that can replicate gene ORF, also used to construct complementary plasmids, were used for the PCR confirmation of wild-type, Δ138, and Δ138-C strains. PCR results of the *cyp138* gene in the wild-type, Δ138 (*cyp138*-knockout), and complementary strains (Δ138-C).

Gene deletion was achieved by overlap extension PCR (Lee et al., 2004), as shown in Figure 1A. Through a two-step PCR process, only the 493-bp fragment of the *Rv0136* coding gene (with a full length of 1,326 bp) was retained. A suicide vector was generated by amplifying the flanking gene of *Rv0136* (*cyp138*) using primer pairs Fa 5′-ACAAGCTT TTCAGACCAGGGAAATGC-3′, Rb 5′-ATGGCTTACGGAACCGAACATCCGACT-3′, Fc 5′-TTCGGTTCC GTAAGCCATCCCCGTTTG-3′, and Rd 5′-ATGCGGCCGC ATCACCGACCACAGCGAC-3′ and cloned into p2NIL by HindIII-NotI restriction sites. Then the hygR, lacZ, and sacB cassettes from pGOAL19 were inserted into the p2NIL-Δ*cyp138* plasmid by Pac I to generate the suicide vector pGNL138. A complementing vector pCM138 was generated by amplifying the gene of *Rv0136*(*cyp138*) using primers 5′-GATTAATTAAGATCGAGGCGACCAGGCC-3′ and 5′-ATC TTAATTAAGGTGACCGAGGTCAGCCC-3′ and cloned into pAPA3 by Pac I restriction site (Brown, 2009). For the PCR of gene sequences with high GC abundance, GC buffer (Takara-Bio, Dalian, China) was used to reduce non-specific amplification.

### 2.3 Gene switching and essentiality testing

The single crossover strain (SCO) was generated by transforming the suicide vector pGNL138 carrying the in-frame deletion into the *Mtb* H37Rv strain. Double crossover strain (DCO) was then generated by transforming the complementing vector pCM138 into SCO, and sucrose with gradient concentrations was used to screen positive strains. DCO was then electroporated with a pUC-Hyg-Int plasmid to switch the complementing vector for essentiality confirmation (Brown, 2009).

### 2.4 Evaluation of bacterial growth status

The growth curves of the wild-type, Δ138, and Δ138-C were measured as previously reported (Penuelas-Urquides et al., 2013). Briefly, the bacterial suspension was diluted using 7H9 medium to OD_600_ = 0.01 and then cultured at 37°C with 5% CO_2_ for 3 weeks. Three biological replicates were measured for each group, and the same volume of suspension was taken and measured at OD_600_.

For growth on defined carbon sources, strains were grown in minimal medium (0.5 g·L^−1^ asparagine, 1.0 g·L^−1^ KH_2_PO_4_, 2.5 g·L^−1^ Na_2_HPO_4_, 50 mg·L^−1^ ferric ammonium citrate, 0.5 g·L^−1^ MgSO_4_·7H_2_O, 0.5 mg·L^−1^ CaCl_2_, 0.1 mg·L^−1^ ZnSO_4_, and 0.05% tyloxapol) containing 0.1% glycerol (v/v) or 0.01% cholesterol (wt/v). Before addition, cholesterol was dissolved to 100 mg·ml^−1^ in a solution of tyloxapol/ethanol (1:1) at 80°C for 30 min (Lee et al., 2013). Bacterial growth under different carbon source conditions was monitored by measuring the optical density at 600 nm of strains on days 0, 7, and 14.

### 2.5 Macrophage infection

The macrophage infection experiment for *Mtb* was performed as previously reported, with slight changes (Hu et al., 2006; Xu et al., 2017). The wild-type, Δ138, and Δ138*-*C strains were grown at 37°C in 7H9 broth for 21 days. The J774A.1 macrophage cell line was cultured in DMEM medium (Gibco, United States), supplemented with 10% fetal bovine serum (FBS) at 37°C with 5% CO_2_. Macrophages were seeded in 24-well culture plates at a density of 4 × 10^5^ cells per well in DMEM medium with 10% FBS and incubated for 16 h at 37°C with 5% CO_2_. Infection of J774A.v1 cells with wild-type, Δ138, and Δ138*-*C strains was performed at a multiplicity of infection (MOI) of 1:1 (bacteria/macrophage). After incubation for 4 h, cells were washed twice with a fresh medium to remove extracellular bacteria. Fresh DMEM medium containing 10% FBS was added per well, and the cells were cultured at 37°C with 5% CO_2_. Macrophages were lysed with 200 μl 0.1% SDS at 37°C for 5 min separately at 0, 1, 3, 5, and 7 days after infection, and a fresh medium was added to terminate lysis. The mixture was diluted to a proper concentration by serial dilutions, and then 100 μl of samples were spread onto the 7H10 culture plate and incubated at 37°C with 5% CO_2_. After 3 weeks, the colonies' numbers of the wild-type, Δ138, and Δ138*-*C strains were counted, respectively.

### 2.6 Scanning electron microscope

The wild-type, Δ138, and Δ138-C strains were cultured in the Middlebrook 7H9 medium supplemented with 0.2% glycerol, 10% OADC, and 0.05% tyloxapol for 14 days. One ml of bacteria cultures were collected by centrifugation at 5,000 × *g* for 5 min, and 1 × PBS buffer was used to wash the collected bacteria. 2.5% glutaraldehyde solution was added, and samples were kept at RT for 1 h, and then at 4°C overnight. The prepared samples were then examined using a scanning electron microscope (SU8020).

### 2.7 Minimum inhibitory concentration tests

The MIC of different antimicrobials against the wild-type, Δ138, and Δ138-C strains were measured using the micro-dilution method. The strains were cultured in 7H9 medium. Antimicrobials including ampicillin, polymyxin B, trimethoxil, actinomycin, rifampicin, isoniazid, ethambutol, streptomycin, levofloxacin, erythromycin, tetracycline, minocycline, D-cycloserine, and vancomycin were included in the test. Results were measured by the microplate alamar blue assay (MABA) and were performed in 96-well black microplates (Collins and Franzblau, 1997). Antimicrobials in gradient concentrations were prepared from 2-fold serial dilutions in the 7H9 medium. The same volume of diluted bacterial samples was transferred to the wells of a 96-well plate to obtain a final density of 5 × 10^5^ CFU·ml^−1^. Three biological replicates were performed for all the groups. Microplates were then incubated at 37°C for 7 days. Alamar blue and 20% Tween-80 were added, and microplates were incubated at 37°C for 1 day. Alamar color will change from blue to purple with bacteria growth. The minimum drug concentration that inhibits bacterial growth is the MIC value.

### 2.8 Protein extraction and TMTsixplex quantitative proteomics analysis

The wild-type and Δ138 strains were cultured as mentioned above. Three biological replicates were prepared, and bacteria of different groups were harvested at the logarithmic (OD_600_ = 0.6) phase. The same amounts of bacteria were collected and suspended in SDC buffer (50 mM NH_4_HCO_3_, 2% sodium deoxycholate, 75 mM NaCl, pH 8.5). Then ultrasonication (3s on, 6s rest, 30%) was used to help lysis. Protein solutions were heated at 95°C for 20 min, and their concentrations were determined using a BCA protein quantification kit. Two hundred μg of proteins from each sample were reduced by 10 mM dithiothreitol (DTT) and digested by trypsin (1:50 (w/w), Promega, Madison, WI, United States) overnight at 37°C. SDC was removed by 1% formic acid (FA), and the sample was desalted by C18 reverse-phase tips (Reprosil-Pur Basic C18, 5 μm, Dr. Maisch GmbH). Peptides were frozen-dried and resuspended in 50 mM HEPES (pH 8.5). Equal amounts of peptides from each sample were used for 6-plex TMT labeling (Thermo Fisher Scientific, United States). Wild-type replicates were labeled with TMT^TM^-126, TMT^TM^-127, and TMT^TM^-128, and Δ138 replicates were labeled with TMT^TM^-129, TMT^TM^-130, and TMT^TM^-131, respectively. Labeled processes were performed by incubating the samples for 1 h at RT (pH = 8.0–8.5). Reactions were quenched by 8 μl of 5% hydroxylamine and incubated for 15 min. Label efficiency was tested, and equal amounts of peptides from different groups were mixed and fractionated using the C18 column (Durashell C18, 3 μm, 150 Å). Peptides were serially eluted by 6%, 9%, 12%, 15%, 18%, 21%, 25%, 30%, 35%, and 50% ACN (50 mM NH_4_CO_3_, pH 10). The fractions were then mixed into seven fractions and vacuum-dried. The peptides were resuspended in water with 0.1% FA and subjected to LC-MS/MS analysis.

The fractions were analyzed on a Nano LC 1200-Orbitrap Fusion Lumos platform with a trap column [ReproSil-Pur 120 C18-AQ (3 μm, Dr. Maisch GmbH, Germany); 20 × 0.05 mm] and a C18 column [ReproSil-Pur 120 C18 (1.9 μm, Dr. Maisch GmbH); 150 × 0.15 mm] at a flow rate of 600 μl·min^−1^. Seven different Nano LC methods with a liner gradient of 15%−95% solvent B (80% acetonitrile with 0.1% FA) over 75 min were used, and data-dependent acquisition with full scans in the 350–1,550 m/z range was carried out using an Orbitrap mass analyzer at a mass resolution of 120,000 at 200 m/z. Parameters were set as follows: cycle time, 3 s; charge, 2–7; intensity threshold, 5.0e^3^; MS2 OT (HCD, collision energy, 38%; isolation window, 0.7; orbitrap resolution, 15,000).

Peptide and protein identification was acquired through Thermo Scientific™ Proteome Discoverer™ version 2.2 (PD2.2). The protein database used for searching was downloaded from UniProt^1^ Protein digestion with two missed cleavages was allowed for each peptide. The search parameters were set as follows: MS accuracy, 10 ppm; MS/MS accuracy, 0.6 Da; dynamic modification (protein terminus) for acetyl; dynamic modification for oxidation; and static modification for carbamidomethyl on cysteine and TMTsixplex were set as fixed modifications. In all cases, the FDR for peptide identification was limited to a maximum of 0.01 by using a decoy database and the percolator algorithm. A hypothetical test (*t*-test) was applied, and proteins with a fold change >1.2 and *p* < 0.05 were considered significantly changed proteins. Functional annotation and clustering enrichment of differentially expressed proteins were conducted using the Gene Ontology (GO) enrichment tool. KEGG pathway enrichment and STRING analysis were also used to find the relationship between proteins. All data have been deposited with the ProteomeXchange Consortium via the PRIDE (Perez-Riverol et al., 2022) partner repository with the dataset identifier PXD045505.

### 2.9 Lipidomic analysis by LipidSearch

The same amounts of bacteria were collected as described in the proteomic analysis. Six biological replicates were prepared for each group. Bacteria were washed with pre-cooled PBS and water. A volume of 240 μl pre-cooled methanol, 200 μl water, and 800 μl MTBE were added before ultrasound in a low-temperature water bath for 20 min. After standing at RT for 30 min, samples were centrifuged at a speed of 14,000 × *g* at 10°C for 15 min. The organic phase was collected and dried. Samples were resuspended in 200 μl of 90% isopropanol/acetonitrile and centrifuged at a speed of 14,000 × *g* for LC-MS/MS analysis.

The samples were separated using the UHPLC Nexera LC-30A system at 45°C with a flow rate of 300 μl·min^−1^. Solvent A is composed of 10 mM ammonium formate and acetonitrile aqueous solution (acetonitrile: water = 6:4, v/v), and solvent B is composed of 10 mM ammonium formate and acetonitrile isopropanol solution (acetonitrile: isopropanol = 1:9, v/v). The gradient elution procedure was set as follows: solvent B was maintained at 30% for 2 min, then solvent B was raised linearly from 30% to 100% in 23 min, and finally, solvent B was maintained at 30% for 10 min. Positive ion and negative ion modes of electrospray ionization (ESI) were used for detection. The samples were analyzed using a Q Exactive plus mass spectrometer (Thermo Fisher Scientific, United States). The ESI source conditions are as follows: for positive: heater temperature, 300°C; sheath gas flow rate, 45 arb; auxiliary gas flow rate,15 arb; sweep gas flow rate, 1 arb; spray voltage, 3.0 KV; capillary temperature, 350°C; S-Lens RF level, 50%; MS1 scan ranges, 200–1,800 m/z. For negative: heater temperature, 300°C; sheath gas flow rate, 45 arb; auxiliary gas flow rate, 15 arb; sweep gas flow rate, 1 arb; spray voltage, 2.5 KV; capillary temperature, 350°C; S-Lens RF level, 60%; MS1 scan ranges, 250–1,800 m/z. In total, 10 fragment patterns (MS2 scan, HCD) are collected after each full scan. MS1 has a resolution of 70,000 at 200 m/z, and MS2 has a resolution of 17,500 at 200 m/z.

The LipidSearch version 4.1 software (Thermo Fisher Scientific, United States) was used for peak identification, lipid identification (secondary identification), peak extraction, peak alignment, and quantification. The main parameters were set as follows: precise tolerance, 5 ppm; product tolerance, 5 ppm; and product ion threshold, 5%. The abundance of lipid molecules with an RSD >30% was deleted. Lipid molecules with missing values >50% in each group were deleted. All the data were normalized by the total peak area. SIMCA-P 14.1 (Umetrics, Umea, Sweden) was used for pattern recognition. After the data were preprocessed by Pareto-scaling, multi-dimensional statistical analysis was performed, including unsupervised PCA analysis and orthogonal partial least squares discriminant analysis (OPLS-DA). One-dimensional statistical analysis was also performed, including the Student's *t-*test and fold change analysis. Volcano diagrams, hierarchical cluster analysis, and correlation analysis were analyzed using the R software.

### 2.10 Lipidomic analysis by MS-LAMP

The LipidSearch version 4.1 software (Thermo Fisher Scientific, USA) was used for peak extraction and quantification. The abundance of lipid molecules with an RSD >30% was deleted. Lipid molecules with missing values >50% in each group were deleted. All the data were normalized by the total peak area. The “Wu Kong” platform (https://www.omicsolution.com/wkomics/main/) (Zhou et al., 2023) was used for multi-dimensional statistical analysis, including Student's *t-*test, PCA, OPLS-DA, and fold change analysis. The data were then interpreted by “*Mtb* LipidDB” of MS-LAMP (Sabareesh and Singh, 2013), and [M+H]+, [M+NH4]+, and [M+Na]+ ions were searched with a mass window range as 0.5.

### 2.11 Targeted proteomics

To confirm the expression of proteins, a targeted proteomics method based on parallel reaction monitoring (PRM) was used to validate the expression differences of proteins as previously reported (Lu et al., 2021a,b). The sample preparation process was the same as that of the DDA method. Unique peptides were chosen to quantify proteins. Raw data were analyzed by Skyline, and the spectral library was built from label-free DDA results. The data were filtered by CV (coefficient of variation) < 30%. Peptides were selected by a *t*-test with a *p*-value < 0.05.

### 2.12 TLC analysis

The extraction and purification of lipids were performed as described previously (Siméone et al., 2010; Janagama et al., 2013). *Mtb* strains were cultured in Middlebrook 7H9 medium supplemented with 0.2% glycerol, 10% OADC, and 0.05% tyloxapol. An additional 0.05 mM propionate was added to study the generation of pthiocerol dimycoserosate (PDIM) and triacylglycerols (TG) under the propionate condition. The bacterial count was kept consistent across all groups, and all *Mtb* strains were harvested by centrifugation at 5,000 × *g* for 5 min and sterilized by adding CHCl_3_/CH_3_OH (1:2, v/v) for 1 day at room temperature. Lipids were extracted with CHCl_3_/CH_3_OH (2:1, v/v) and CHCl_3_/CH_3_OH (1:1, v/v) for 1 h at room temperature, respectively. Then, it was washed twice with water and dried before analysis. After resuspension in an equal volume of CHCl_3_, the extracted lipids were analyzed by TLC. Equivalent amounts of lipids from each extraction were spotted onto silica gel G60 plates (20 × 20 cm, Shanghai Titan Scientific Co., Ltd.) and separated with petroleum ether/diethyl ether (90:10, v/v) for PDIM and TG analysis. PDIM and TG were visualized by spraying the plates with 10% phosphomolybdic acid in ethanol, followed by heating at 120°C.

## 3 Results

### 3.1 Essentiality testing and construction of knockout and complementary strains

Gene knockout was performed using the overlap extension PCR method shown in Figure 1A, and the gene non-essentiality of *cyp138* was confirmed by gene switching. A two-step homologous recombination procedure was combined with complemented *cyp138* encoded on an integrated vector derived from the L5 mycobacteriophage to generate the merodiploid strain. The resident integrated vector was removed and replaced by an alternative version carrying a different antibiotic selection marker for gene switching. DCO with complemented *cyp138* was obtained, and *cyp138* was proved to be a non-essential gene. In the meantime, the complementary strain was also obtained. Primers used to construct the complementary plasmid were used for the PCR confirmation of wild-type, Δ138, and Δ138-C strains (Figure 1B).

### 3.2 Evaluation of growth status in different culture conditions and infection ability in macrophages

**Figure 2:**
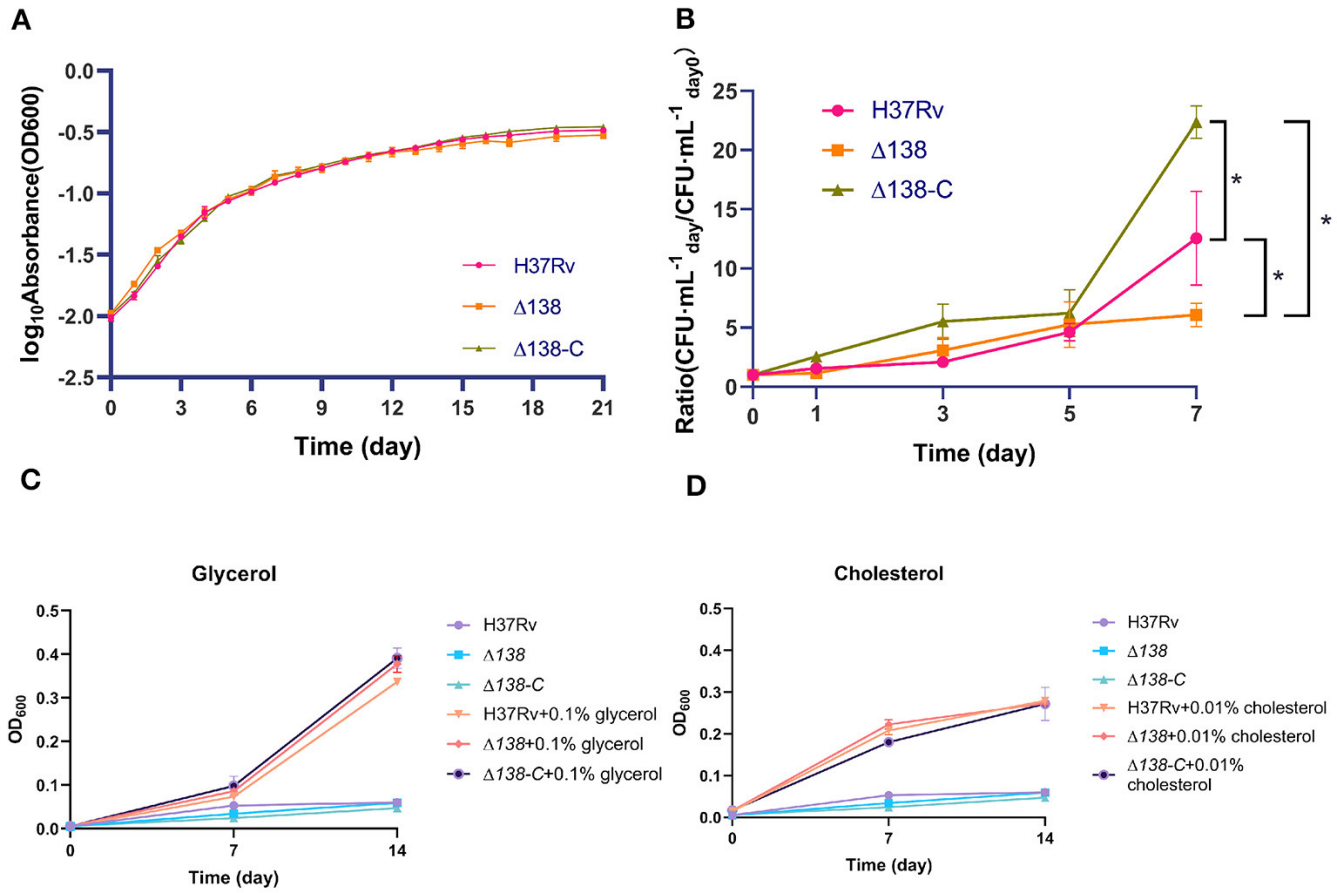
**(A)** The growth status of the wild-type, Δ138, and Δ138-C strains in the 7H9 medium supplemented with 0.2% (v/v) glycerol, 0.05% (v/v) Tween 80, and 10% (v/v) OADC at 37°C. The Δ138-C was cultured in the 7H9 broth mentioned above, supplemented with an additional 10 μg·ml^−1^ gentamicin, with three replicates per group, and the bacteria density was monitored at OD_600_. A two-way ANOVA analysis was used for the statistical analysis among groups and no significant difference was observed; **(B)** intracellular counts of the wild-type, Δ138, and Δ138-C strains in macrophages after infection by culturing on the 7H10 culture plate supplemented with 10% OADC at 37°C for 14 days with three replicates per group, and a rank-sum test was used for the statistical analysis; **(C, D)** the indicated strains were cultured in defined medium containing 0.1% glycerol **(C)** or 0.01% cholesterol **(D)** as the sole carbon source.

The growth status of H37Rv strains was evaluated by monitoring the absorbance of bacterial culture at OD_600_. As shown in Figure 2A, the absorbance values of the wild-type, Δ138, and Δ138-C increased continuously. Similar growth curves were observed among the wild-type, Δ138, and Δ138*-*C groups.

As glycerol and cholesterol are important carbon and energy sources of *Mtb* (Pandey and Sassetti, 2008; Martinez et al., 2023), and CYP125 was previously proved to be essential for cholesterol catabolism (Johnston et al., 2012), we performed the sole carbon source experiments to explore whether CYP138 was involved in the carbon source utilization. As shown in Figures 2C, D, no growth was observed without the addition of a carbon source, and there was no significant difference in the growth rate of the wild-type H37Rv, Δ138, and Δ138-C strains, whether supplementing with 0.1% glycerol or 0.01% cholesterol in the medium.

Compared with the increasing trend of intracellular counts of the wild-type and Δ138-C, that of the Δ138 in macrophages was relatively gentler (Figure 2B). After 7 days of infection, the CFU number of the Δ138 collected from cells was significantly lower than that of the wild-type and Δ138-C in macrophages (*p* < 0.05).

### 3.3 Evaluation of antimicrobial susceptibility

To further investigate the effect of *cyp138*-knockout on the susceptibility of *Mtb* to antibiotics, 14 antimicrobial drugs (as shown in Table 1) were selected for MIC tests of the wild-type, Δ138, and Δ138-C strains, respectively. Compared with the wild-type and Δ138-C strains, Δ138 showed similar susceptibility to actinomycin, isoniazid, ethambutol, streptomycin, levofloxacin, tetracycline, erythromycin, D-cycloserine, and minocycline. It is worth noting that compared to the wild-type strain, the MIC values of ampicillin and vancomycin against the Δ138 strain decreased by eight times and four times, respectively, while the MIC values of polymyxin B against the Δ138 strain increased by four times.

**Table 1:** Results of MIC tests of the wild-type, Δ138, and Δ138-C strains.

	**MIC (**μ**g**·**ml**^**−1**^**)**		
**Strains**	**Wild type**	Δ**138**	Δ**138-C**
Polymyxin B	512	2,048	1,024
Ampicillin	128	16	128
Actinomycin	1,024	1,024	1,024
Trimethoprim	1,024	512	1,024
Rifampicin	0.125	0.0625	0.125
Isoniazid	0.0625	0.0625	0.0625
Ethambutol	0.0625	0.0625	0.0625
Streptomycin	0.5	0.5	0.5
Levofloxacin	0.125	0.125	0.125
Tetracycline	16	16	16
Erythromycin	512	512	1,024
D-cycloserine	4	4	4
Vancomycin	512	128	512
Minocycline	4	4	4

### 3.4 Effects of *cyp138-*knockout on protein expression

**Figure 3:**
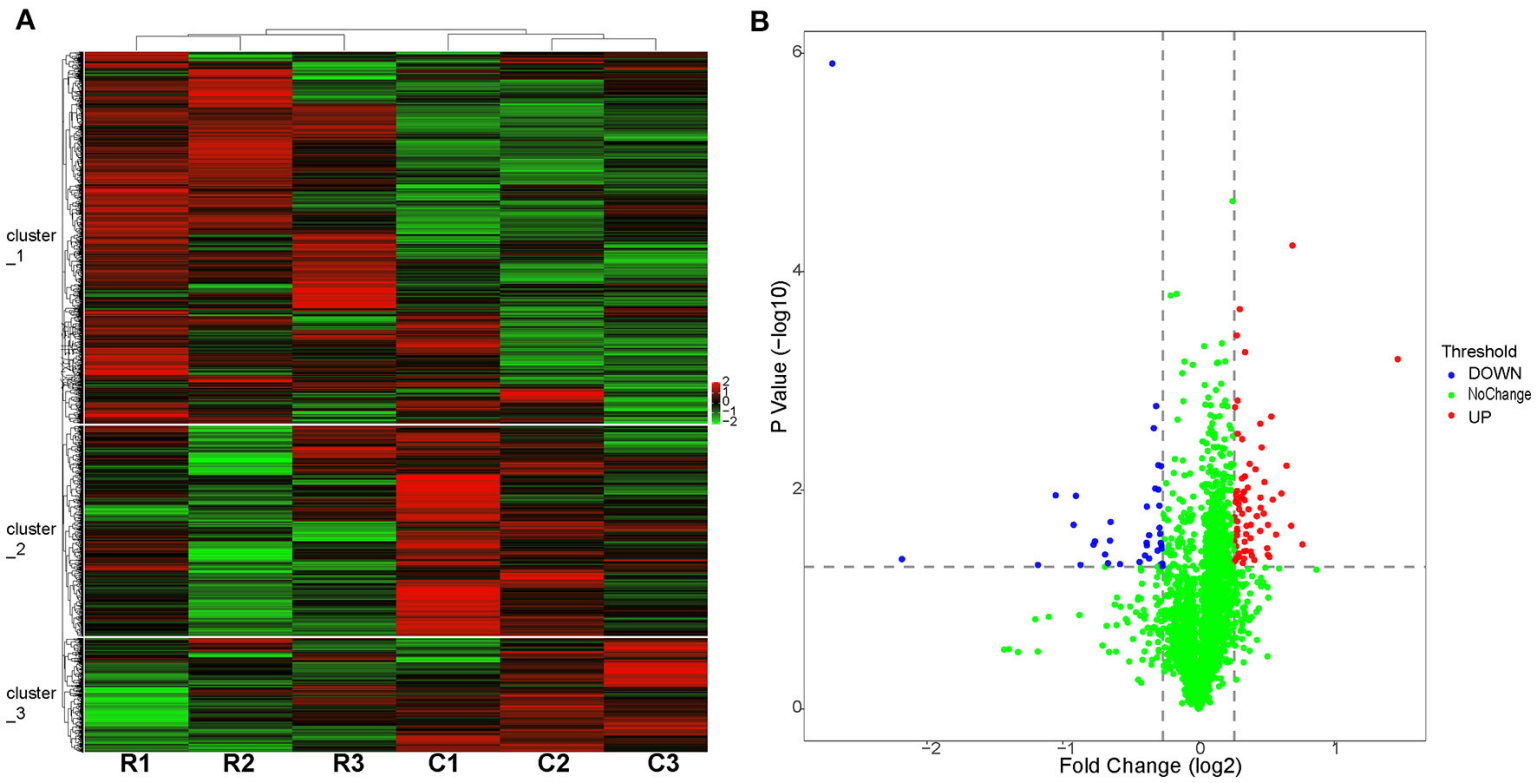
Proteome profiles of the wild-type or *cyp138-*knockout *Mtb* H37Rv. **(A)** Hierarchical clustering of proteomes with three biological replicates. Red represents the highest relative expression, and green represents the lowest. C1, C2, and C3 (the wide-type); R1, R2, and R3 (the *cyp138-*knockout). **(B)** Volcano distribution of proteins divided by *p* < 0.05 and fold change >1.2; red represents downregulated, and blue represents upregulated.

To identify proteins that may be potentially associated with CYP138, we compared the proteome of the wild-type with the Δ138 strains using a 6-plex TMT-labeling-based quantitative proteomic approach. Bacteria cells, harvested at their logarithmic phases in three biological replicates, were analyzed by Nano LC-MS/MS. In total, 2,726 bacterial proteins were identified in both groups. As shown in Figure 3A, good stability and reproducibility were observed in both groups. Based on *p* < 0.05 and fold change >1.2, the expression levels of 109 proteins were finally identified to be significantly different in the Δ138 group. Among these, 37 proteins exhibited decreased accumulation and 72 proteins exhibited increased accumulation (as shown in Figure 3B and Supplementary Table S1).

**Figure 4:**
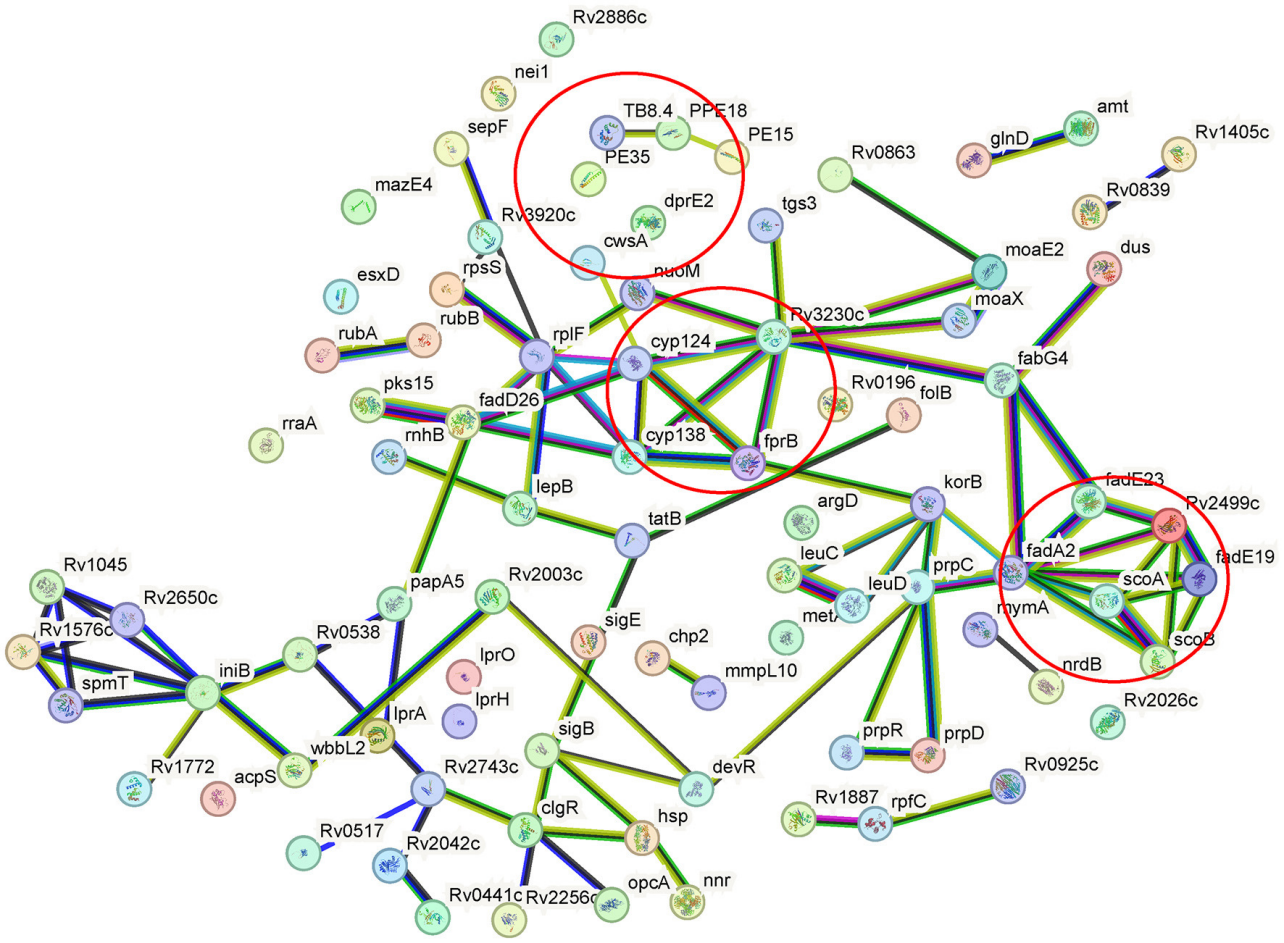
Protein–protein interaction networks of differentially expressed proteins of the wild-type or *cyp138-*knockout *Mtb* H37Rv. Nodes represent proteins, while edges represent protein–protein interactions. Colored nodes represent clusters. Colored edges: known interactions from: teal, curated databases; magenta, experimentally determined; predicted interactions: green, gene neighborhood; red, gene fusions; blue, gene co-occurrence; others: yellow, text mining; black, coexpression; purple, protein homology. Partial KEGG pathways were marked by a circle.

By using GO annotation enrichment analysis, as shown in Supplementary Figure S2, the expression levels of proteins related to oxidation-reduction process, pathogenesis, growth of symbiont in the host cell, valine catabolic process response to hypoxia, etc. in biological process (BP); plasma membrane, an integral component of membrane, extracellular region, cytosol, cytoplasm, etc. in cellular component (CC); and DNA binding, metal ion binding, iron ion binding, flavin adenine dinucleotide binding, etc. in molecular function (MF) were observed to be significantly changed. According to the KEGG pathway enrichment results shown in Supplementary Figure S3, 20 significantly expressed proteins were involved in metabolic pathways. The expression levels of prpC, leuD, leuC, and metA in the biosynthesis of amino acids pathway were all increased, and that of argD decreased. FadA2, prpC, argD, KorB, and MymA were involved in microbial metabolism in diverse environmental pathways. FadA2, prpC, and KorB also belonged to the carbon metabolism pathway. FadA2, fadE19, scoB, scoA, and fadE23 were involved in the valine, leucine, and isoleucine degradation pathways. FabG4, moaE2, moaX, and folB were involved in the biosynthesis of the cofactors pathway. FadA2, KorB, scoB, and scoA were involved in the butanoate metabolism pathway. FabG4, fadA2, fadE23, and desA3 (Rv3230c) were involved in the fatty acid metabolism pathway, and the expression levels of all these proteins were increased. FadA2 and FadE23 also belong to the fatty acid degradation pathway. PrpC, argD, leuD, and leuC belonged to the 2-oxocarboxylic acid metabolism pathway. LeuD and leuC also belonged to the C5-branched dibasic acid metabolism and valine, leucine, and isoleucine biosynthesis pathway. FadA2, glinD, and devR belonged to the two-component system pathway. MoaE2, moaX, and folB belonged to the folate biosynthesis pathway. MoaE2 and moaX were also related to the sulfur relay system pathway. Protein–protein interaction analysis by STRING (Figure 4) revealed that clusters were found, and core proteins such as prpC, fadA2, fadG4, fadE23, scoB, desA3, cyp138, fadD26, Rv2499c, and sigB were acquired, indicating the important roles they played in the network.

### 3.5 Differences in lipid metabolism annotated by LipidSearch

To investigate the effects of the *cyp138*-knockout on the production of lipids, lipidomics based on LC-MS/MS were performed. A total of 241 lipid species among 16 lipid classes were identified by LipidSearch in total. The PCA results of the control and gene knockout groups are shown in Supplementary Table S2 and Supplementary Figure S4A. In summary, the data of the samples in the same group were stable and reliable, and differences between the groups could be observed. The data acquired were analyzed using OPLS-DA. It can be seen that the OPLS-DA model can distinguish two sets of samples (Supplementary Figure S4B). According to the VIP values (variable importance for the projection) obtained by the OPLS-DA model, differentially produced lipids with biological significance are discovered. Volcano plots are shown in Supplementary Figure S5A. In this experiment, lipids with VIP > 1 and *p-*value < 0.05 were selected as the lipids with significant differences, and 15 lipids belonging to the TG class were acquired, as shown in Table 2 and Supplementary Figure S5B.

**Table 2:** Lipids with significant differences affected by *cyp138*-knockout and selected by VIP>1 and *p*-value < 0.05.

**Lipid Ion**	**Class**	**Fatty Acid**	**Ion Formula**	**CalcMz**	**RT(min)**	**VIP**	**Fold change**	***P*-Value**
TG(16:1/18:2/18:3)+NH4	TG	(16:1/18:2/18:3)	C55 H98 O6 N1	868.7388655	18.50068935	1.51277	0.575183456	0.027456008
TG(20:0/16:0/18:1)+NH4	TG	(20:0/16:0/18:1)	C57 H112 O6 N1	906.8484155	23.65975995	1.69167	1.443537104	0.031452067
TG(18:0/18:1/18:2)+NH4	TG	(18:0/18:1/18:2)	C57 H108 O6 N1	902.8171155	21.50437738	4.63207	1.550719532	0.024463195
TG(16:0/18:1/22:1)+NH4	TG	(16:0/18:1/22:1)	C59 H114 O6 N1	932.8640655	23.03547947	1.78592	1.79253124	0.002596323
TG(20:1/18:1/18:1)+NH4	TG	(20:1/18:1/18:1)	C59 H112 O6 N1	930.8484155	22.30441518	2.94074	1.868213222	0.00336453
TG(20:1/18:1/18:2)+NH4	TG	(20:1/18:1/18:2)	C59 H110 O6 N1	928.8327655	21.51308038	2.96771	1.991436574	0.008196598
TG(26:0/16:0/16:0)+NH4	TG	(26:0/16:0/16:0)	C61 H122 O6 N1	964.9266655	25.05756867	1.48333	1.562056334	0.023229385
TG(16:0/18:1/24:1)+NH4	TG	(16:0/18:1/24:1)	C61 H118 O6 N1	960.8953655	23.73997583	1.43663	1.794524514	0.005769944
TG(18:1/18:1/22:1)+NH4	TG	(18:1/18:1/22:1)	C61 H116 O6 N1	958.8797155	23.08435855	1.06999	1.76465305	0.016931395
TG(26:0/16:0/18:1)+NH4	TG	(26:0/16:0/18:1)	C63 H124 O6 N1	990.9423155	24.56273872	2.00241	1.523469872	0.03224491
TG(26:0/16:1/18:1)+NH4	TG	(26:0/16:1/18:1)	C63 H122 O6 N1	988.9266655	24.15275172	1.65893	1.538378437	0.012527172
TG(18:1/18:1/24:1)+NH4	TG	(18:1/18:1/24:1)	C63 H120 O6 N1	986.9110155	23.72456073	1.20174	1.673546326	0.025807869
TG(26:0/18:1/18:2)+NH4	TG	(26:0/18:1/18:2)	C65 H124 O6 N1	1014.942315	24.24900233	1.4178	1.598777179	0.013215417
TG(28:0/16:0/24:0)+NH4	TG	(28:0/16:0/24:0)	C71 H142 O6 N1	1105.083165	26.56641218	1.28293	2.766283949	0.003271675
TG(28:0/18:1/24:0)+NH4	TG	(28:0/18:1/24:0)	C73 H144 O6 N1	1131.098815	26.56063782	1.22111	1.865395521	0.001405548

### 3.6 Differences in lipid metabolism annotated by *Mtb* LipidDB (MS-LAMP)

**Figure 5:**
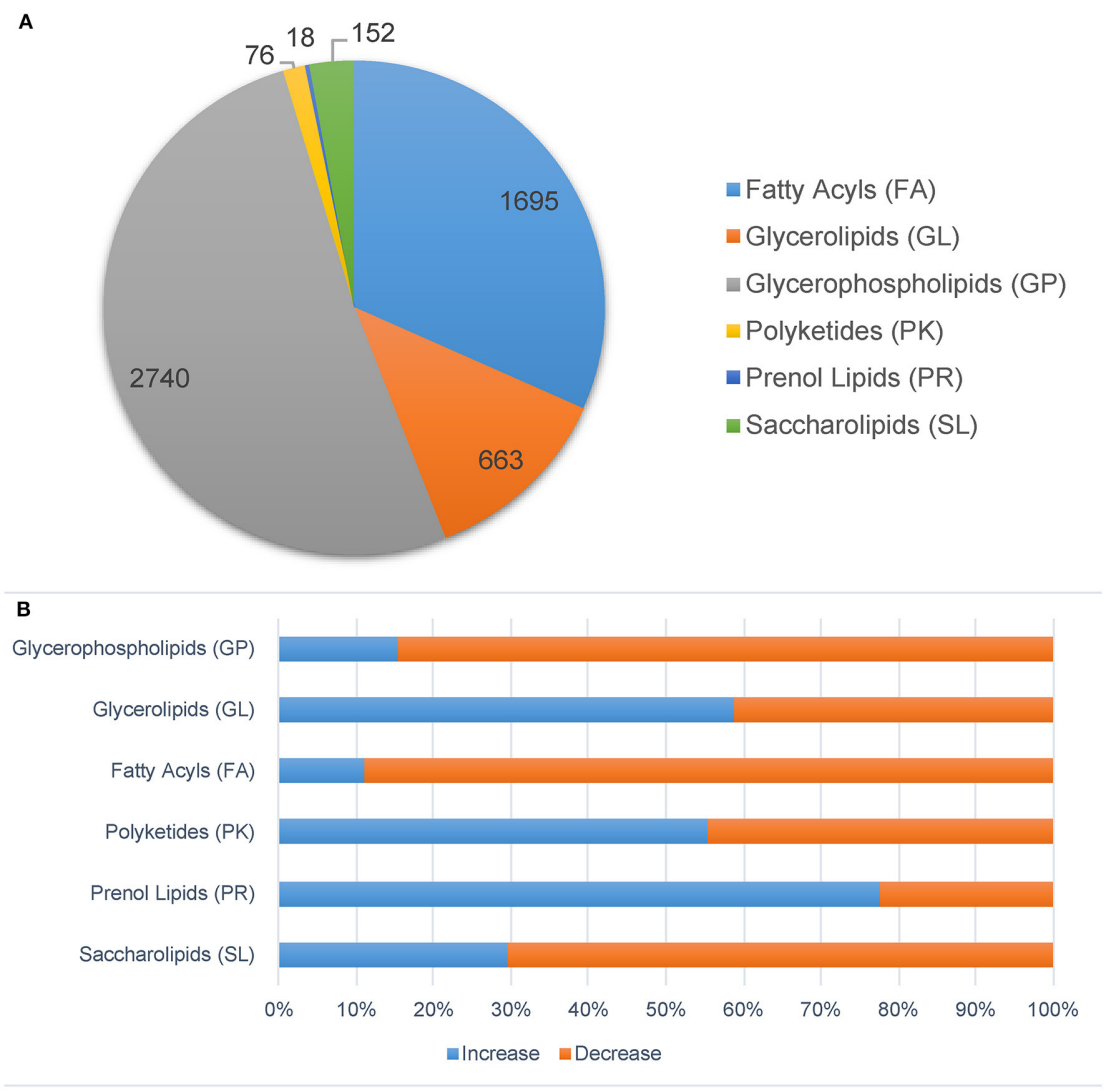
Significantly changed lipids molecules in the *cyp138-*knockout *Mtb* H37Rv filtered by *p* < 0.05, VIP > 1, and fold change > 1.5. Ions were annotated by MS-LAMP. **(A)** Lipid categories. **(B)** The increased and decreased molecules numbers in each category.

We also analyzed the lipidomic data with another database, *Mtb* LipidDB (Sartain et al., 2011), reported by Sartain et al. through *Mtb* Lipidome MS-LAMP. Only data acquired in positive mode were analyzed. In total, 20,911 ions were identified and further analyzed by Student's *t*-test and OPLS-DA (Supplementary Figure S6). After filtering by VIP > 1, *p* < 0.05, and fold change > 1.5, 1,814 ions were identified as significantly changed lipid molecules. These ions were then annotated by *Mtb* LipidDB through MS-LAMP, and 5,344 lipid molecules were matched with a mass window range of 0.5 (Supplementary Table S3). As shown in Figure 5A, glycerophospholipids (GP), fatty acyls (FA), and glycerolipids (GL) have the highest quantity of ions. The levels of most molecules in the FA and GP groups decreased in the Δ138 groups, while the levels of more than half of the GL molecules increased (shown in Figure 5B). Most of these lipids in the FA and GP groups were important molecules of membrane structure.

### 3.7 Verification of targeted proteins

To verify the differently expressed proteins in the wild-type, Δ138, and Δ138-C strains separately, targeted proteomics based on the PRM approach was applied. In total, we reliably quantified two proteins with six peptides (Supplementary Table S4) as significantly expressed proteins filtered by *p* < 0.05 and fold change > 1.5.

### 3.8 Verification of lipids composition by TLC analysis

**Figure 6:**
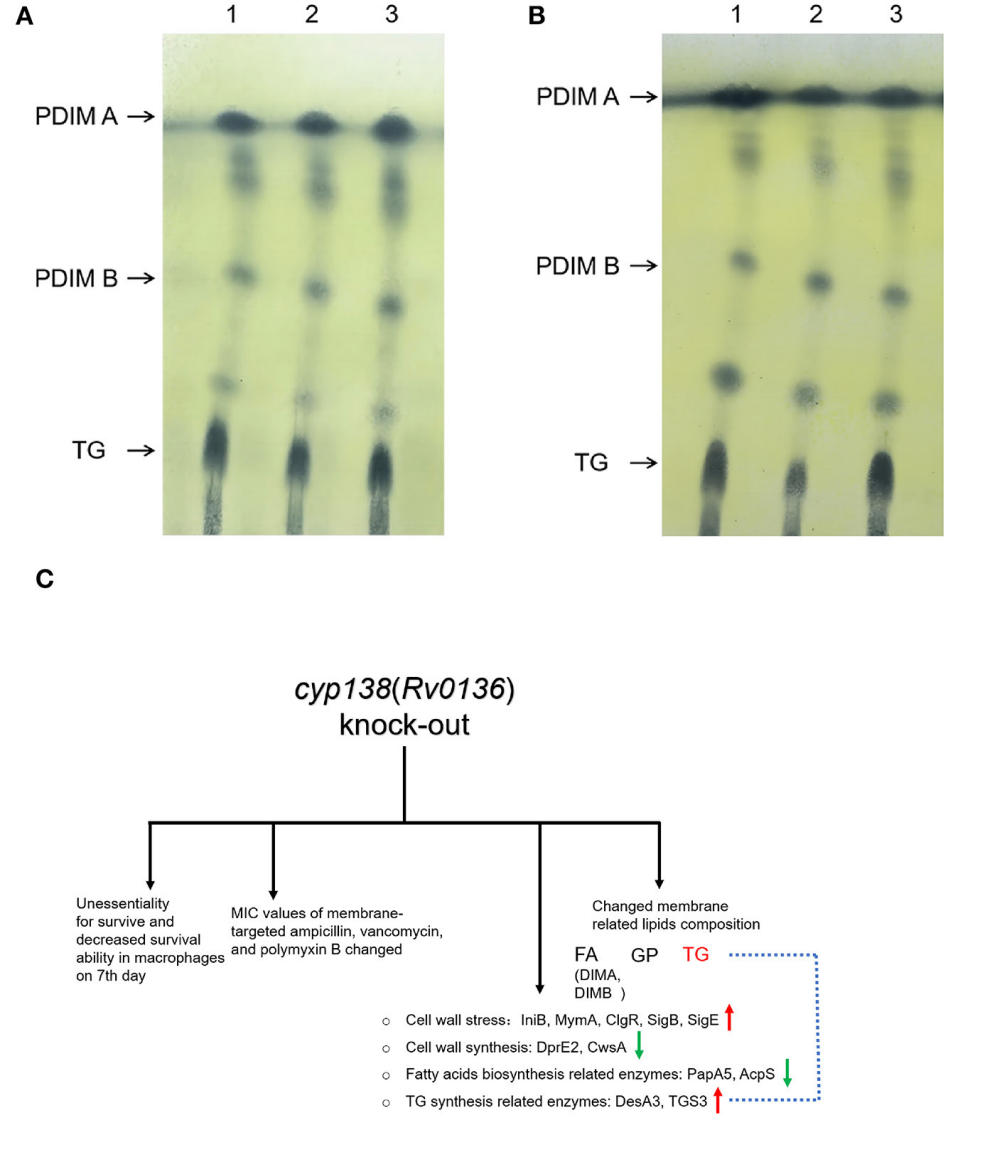
TLC analysis of PDIMs and TG extracted from the wild-type H37Rv, Δ138, and Δ138-C strains. **(A)** The strains were cultured in 7H9 medium. **(B)** The strains were cultured in 7H9 medium supplemented with 0.05 mM propionate. Lane 1, the wild-type H37Rv strain; Lane 2, the Δ138 strain; Lane 3, the Δ138-C strain. **(C)** A general view of the effects of *cyp138-*knockout on *Mtb* H37Rv.

TLC analysis was performed to verify the influence of the *cyp138*-knockout on the lipid composition. As shown in Figures 6A, B, similar amounts of TG were observed in the Δ*138* strain compared to those of wild-type and Δ138-C strains, and an obvious reduction was observed in the culture group with additional propionate supplementation. No significant changes were observed for the levels of PDIMA and PDIMB in the regular culture conditions and the propionate group.

## 4 Discussion

Recently, much more attention has been drawn to the CYPs of *Mtb* due to their unexpected number and important function in cell survival, virulence, and metabolism (Ortega Ugalde et al., 2019). Some of them, such as CYP121 and CYP125, were developed as promising anti-*Mtb* drug targets. However, there is little or no research for almost half of the CYPs in *Mtb*, and CYP138 is one of them. In our study, preliminary explorations on the function of CYP138 were conducted by comparing several properties of the *cyp138-*knockout and wild-type strains.

In this study, we successfully constructed a *cyp138-*knockout strain by homologous recombination and confirmed *cyp138* as a non-essential gene for *in vitro* growth for the first time. Initially, by comparison of the growth curves and macrophage infection status among the wild-type, Δ138, and Δ138-C strains, we found that although there is no significant difference in the 21-day growth *in vitro* among the three strains, the intracellular Δ138 bacterial count on the 7^th^ day after macrophage infection was significantly lower than that of wild-type and Δ138-C strains. Thus, we speculated that *cyp138* might play an important role in maintaining the normal growth and replication of bacteria in the later cellular infection process. The growth experiments on medium using glycerol and cholesterol as the sole carbon sources proved that the knockout of *cyp138* does not affect the utilization of glycerol and cholesterol. SEM results provided us with no changes in bacterial surface morphology (Supplementary Figure S7).

To further investigate the effects of *cyp138*-knockout on the susceptibility of *Mtb* to antimicrobial drugs, 14 antibiotics were used for the MIC test. Interestingly, compared to the wild-type strain, the susceptibility of the Δ138 strain to ampicillin and vancomycin increased. Studies have reported that the action mechanism of ampicillin is binding to primary receptors called membrane-bound penicillin-binding proteins (PBPs) and preventing the late stages of peptidoglycan synthesis in the cell wall (Peechakara et al., 2021). The action mechanism of vancomycin is to inhibit the polymerization of peptidoglycans in the bacterial cell wall (Patel et al., 2021). According to the cell wall structure of *Mtb* reported (Batt et al., 2020), the peptidoglycan layer is located inside the arabinan and mycolic acid layers. The exposure of the peptidoglycan layer, which is the target of ampicillin and vancomycin, indicates damage to the cell wall structure, especially the arabinan and mycolic acid layers. Besides, compared with the wild-type strain, the Δ138 strain had a reduced susceptibility to polymyxin B, which may be due to the loss of some targets or changes in membrane components. Interestingly, ampicillin and vancomycin were not on the list of commonly used anti-TB drugs in clinical practice. The increased susceptibility of the Δ138 strain to them indicated that the absence of *cyp138* can promote the possible application of these drug classes in the fight against TB.

A quantitative proteomics method based on the TMTsixplex-labeled technique was used to discover the differences in proteomes between the wild-type and Δ138 strains. Among the 72 proteins with increased expression levels, fadA2 is involved in 17 pathways, indicating its basic and important roles in metabolism as an acetyl-CoA acyltransferase. Given the increased expression level of fabG4, fadA2, fadE23, and desA3, which are associated with the fatty acid metabolism pathway (as shown in Supplementary Figure S8), fatty acid biosynthesis, elongation, and beta-oxidation would be promoted. PrpR, prpC, and prpD are key enzymes of methylcitrate, which are essential for the growth of mycobacteria on propionate as a sole carbon source *in vitro*. The increased expression of these enzymes also indicated the increased production of propionyl-CoA, the product of odd-chain-length fatty acid metabolism (Masiewicz et al., 2012). MmpL10 is likely responsible for the translocation of diacyl trehalose (DAT) and the biosynthesis of pentacyl trehalose (PAT) (Bailo et al., 2015). Chp2 is essential for the final steps of PAT biosynthesis and is regulated by MmpL10 (Touchette et al., 2015b). The increased expression of MmpL10 and Chp2 might indicate the increased biosynthesis of PAT. Isopropylmalate isomerase (IPMI), a complex of two subunits, namely, LeuC and LeuD, is an enzyme of the leucine biosynthesis pathway, which is absent in humans (Manikandan et al., 2011). The increased expression of LeuC and LeuD indicates an increased level of leucine. The isoniazid-induced protein IniB was reported to respond to cell wall stress (Alland et al., 2000). MymA, also a target of isoniazid, was reported to be required for maintaining the appropriate mycolic acid composition and permeability of *Mtb* on its exposure to acidic pH (Singh et al., 2005). The increased expression of IniB and MymA represents the changes in the cell wall. The expression level of stress-regulated proteins such as sigB and sigE also responds to surface stress (Kundu and Basu, 2021). A previous study reported that over-expression of ClgR also indicated threats to the stability of the cell membrane (Sawicki et al., 2018). Nnr, FprB, and CYP124 are all electron transfer chain-related proteins. As shown in Figure 4, cyp124, FprB, and desA3 showed strong connections with each other, and all of these proteins showed increased levels in the Δ138 strain, indicating the possibility of functional correlation or compensation between them and *cyp*138. Among the 37 proteins with decreased expression levels, fadE19, OXCT A (scoA), and OXCT B (scoB) are involved in the valine, leucine, and isoleucine degradation pathways, while OXCT A (scoA) and OXCT B (scoB) are involved in the butanoate metabolism pathway. Acetylornithine aminotransferase (ACOAT) and the 2-oxoglutarate oxidoreductase subunit KorB are involved in several pathways related to metabolism. DNA-binding transcriptional activator DevR/DosR was reported to participate in the virulence, dormancy adaptation, and antibiotic tolerance mechanisms of *Mtb* (Sharma et al., 2021).

Among these significantly expressed proteins, 48 membrane-related (cell wall, plasma membrane, cell surface, integral component of plasma membrane, integral component of membrane, cell surface, cell outer membrane, etc.) proteins were acquired, while the expression level of 18 membrane-related proteins increased and that of 30 membrane-related proteins decreased. Among the membrane-related proteins with reduced expression levels, DprE2 (P9WGS9) is required for the synthesis of cell-wall arabinans (Manina et al., 2010), and CwsA (P9WJF3) is involved in peptidoglycan synthesis and cell shape determination (Plocinski et al., 2012). The decreased expression levels of these two proteins will affect the cell wall composition and explain the MIC results to some extent. In addition, the decreased expression level of cell division protein SepF (P9WGJ5) will affect cell division and ultimately affect cell proliferation (Gupta et al., 2018). The expression levels of membrane proteins related to immunity, such as LprA (P9WK55), LprH (P9WK43), PPE18, PE35, and TB8.4, all decreased (Skerry et al., 2016).

*Mtb* is abnormally rich in lipids, which are mainly distributed on the cell envelope. TG (also known as TAG) and PDIM (also known as DIMs) were proven to be important components of the *Mtb* membrane (Ortalo-Magné et al., 1996; Daffé and Marrakchi, 2019). TG serves as a dependable, long-term energy source, which is associated with the long-term persistence of *Mtb* (Maurya et al., 2018). According to the omics results, the quantity of 15 TG molecules and TG-related proteins such as desA3 and TGS3 changed. DesA3 is a membrane-bound stearoyl-CoA delta (9)-desaturase that produces oleic acid, a precursor of mycobacterial membrane phospholipids and TG (Chang and Fox, 2006). Triacylglycerol synthase TGS3 is an enzyme that can synthesize TG (Supplementary Figure S9). The levels of TG molecules changed in the Δ138 strain according to the lipidomic data, which might not be apparent in the TLC image as TLC is a relatively rough separation method. The addition of propionate makes this change more pronounced (Figures 6A, B). However, identifying the specific step at which TG synthesis inhibition occurs due to the cyp138-knockout may necessitate additional research. Studies have proven that the addition of a low concentration of propionate can promote the levels of PDIM (Mulholland et al., 2023). Our proteomics results showed that the levels of PapA5 and fadD26, both critical enzymes in PDIM biosynthesis (Onwueme et al., 2005; Touchette et al., 2015a), decreased in the Δ138 strain. The levels of 97% of PDIM molecules were decreased in the lipidomic data shown in Supplementary Table S3. However, no obvious changes in PDIM were observed in the subsequent TLC analysis. Therefore, given the crucial role of TG in cell membrane integrity and long-term cell viability, variations in its content may account for the observed differences in macrophage infection on day 7, as well as changes in cell wall composition and susceptibility to several antibiotics.

Besides, by being reannotated by MS-LAMP, the levels of diacylglycerols (DG) and monoacylglycerols (MG) molecules in the Δ138 group were also changed. GP, like TG, has glycerol as its basic skeleton. For the Δ138 strain, the levels of most molecules in the GP group decreased. PIM, which belongs to GP, serves as the anchor for the biologically important lipoglycans lipoarabinomannan and lipomannan (Pitarque et al., 2008). The level changes of PIM will influence the membrane structure and function. In addition, the changed level of DAT confirmed the increased expression level of MmpL10 and Chp2 and indicated the increased biosynthesis of DAT and PAT. Consistent with the fatty acid metabolism-related proteome dataset, the levels of most FA ions changed, such as MA, GMM, TMM, and PDIM, indicating changes in membrane composition. However, due to the incompleteness of the *Mtb* lipidomic spectrum library, the same ion was matched with multiple molecules by MS-LAMP. With the development of technology, the *Mtb* lipid and KEGG pathway libraries will become more complete, which will improve the clarification of gene function.

## 5 Conclusion

As shown in Figure 6C, the knockout of the *cyp138* gene leads to decreased survival ability within macrophages on the 7^th^ day, changes in susceptibilities to membrane-targeted ampicillin, vancomycin, and polymyxin B, changed levels of membrane-related and lipids metabolism-related proteins, as well as membrane-related lipids composition, especially TG. Besides, it is worth noting that the deletion of *cyp138* significantly improved the susceptibility of the pathogen to ampicillin and vancomycin, which gives us a hint that the development of *cyp138* inhibitors may promote the potential use of β-lactams and glycopeptides in anti-TB treatment. In addition, how CYP138 participates in and affects the synthesis and metabolism of TG and how propionate promotes this effect is still not clear to us. More work needs to be done to further reveal the function, the substrate, and the redox partner of the CYP enzyme.

## Data availability statement

The datasets presented in this study can be found in online repositories and Supplementary material. The names of the repository/repositories and accession number(s) can be found in the article.

## Author contributions

YunL: Conceptualization, Funding acquisition, Methodology, Software, Visualization, Writing—original draft. HC: Formal analysis, Methodology, Validation, Writing—original draft. ZS: Methodology, Validation, Writing—original draft. LS: Software, Writing—review & editing. CL: Supervision, Writing—review & editing. YuL: Project administration, Resources, Writing—review & editing. XYo: Funding acquisition, Project administration, Writing—review & editing. XYa: Conceptualization, Funding acquisition, Project administration, Writing—review & editing.

## Footnotes

^1^ https://www.uniprot.org/
