# Supplementary material for: Deletion of the Mycobacterium tuberculosis cyp138 gene leads to changes in membrane-related lipid composition and antibiotic susceptibility
Source: Front Microbiol. 2024 Mar 25;15:1301204. doi: 10.3389/fmicb.2024.1301204 (PMC10999552; doi:10.3389/fmicb.2024.1301204)
Supplement: Supplementary file 1 [file Data_Sheet_1.zip › Supplementary Figure S1.DOCX]

Supplementary Material

**
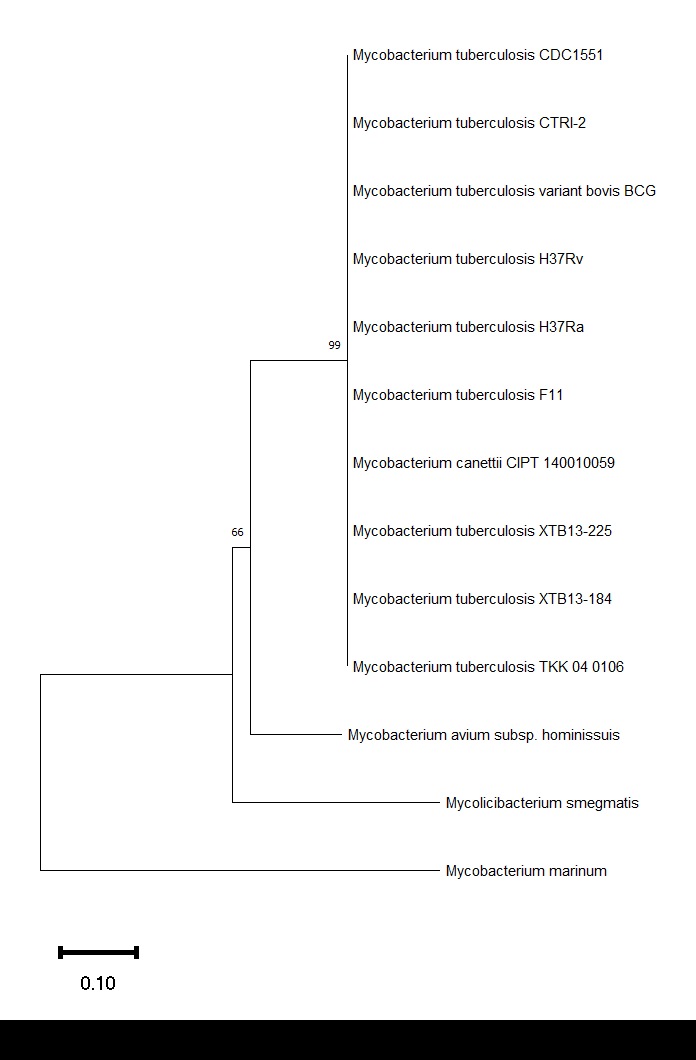
**

**Supplementary Figure S1.** Phylogenetic tree analysis of the amino acid sequences of CYP138.
