# Supplementary material for: Deletion of the Mycobacterium tuberculosis cyp138 gene leads to changes in membrane-related lipid composition and antibiotic susceptibility
Source: Front Microbiol. 2024 Mar 25;15:1301204. doi: 10.3389/fmicb.2024.1301204 (PMC10999552; doi:10.3389/fmicb.2024.1301204)
Supplement: Supplementary file 1 [file Data_Sheet_1.zip › Supplementary Figure S2.DOCX]

Supplementary Material


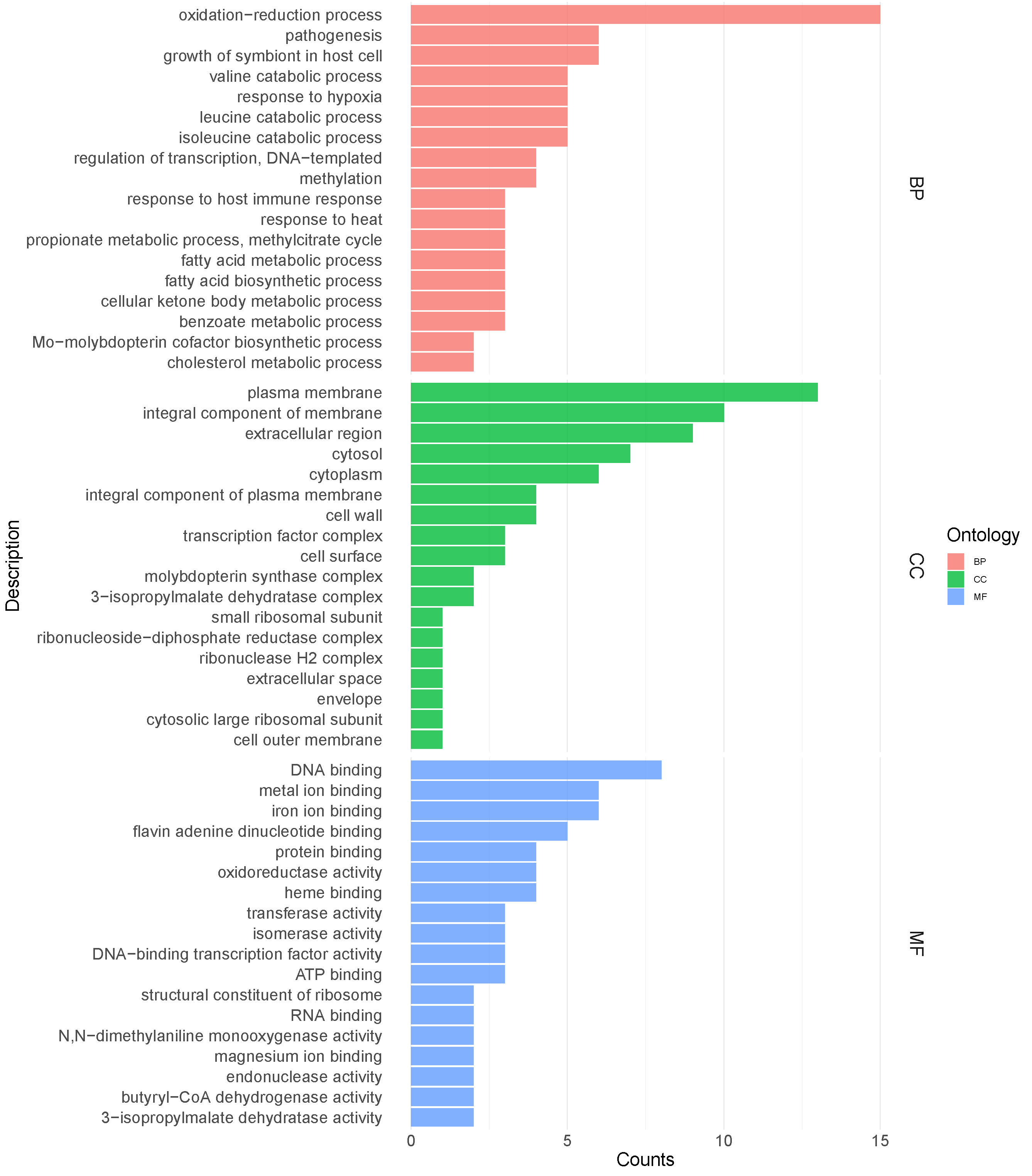


**Supplementary Figure S2.** GO annotation enrichment analysis results of significantly differentially expressed proteins when cyp138 was knocked out from *Mtb* H37Rv, filtered by p < 0.05 and fold change > 1.2.
