# Supplementary material for: Deletion of the Mycobacterium tuberculosis cyp138 gene leads to changes in membrane-related lipid composition and antibiotic susceptibility
Source: Front Microbiol. 2024 Mar 25;15:1301204. doi: 10.3389/fmicb.2024.1301204 (PMC10999552; doi:10.3389/fmicb.2024.1301204)
Supplement: Supplementary file 1 [file Data_Sheet_1.zip › Supplementary Figure S5.DOCX]

Supplementary Material


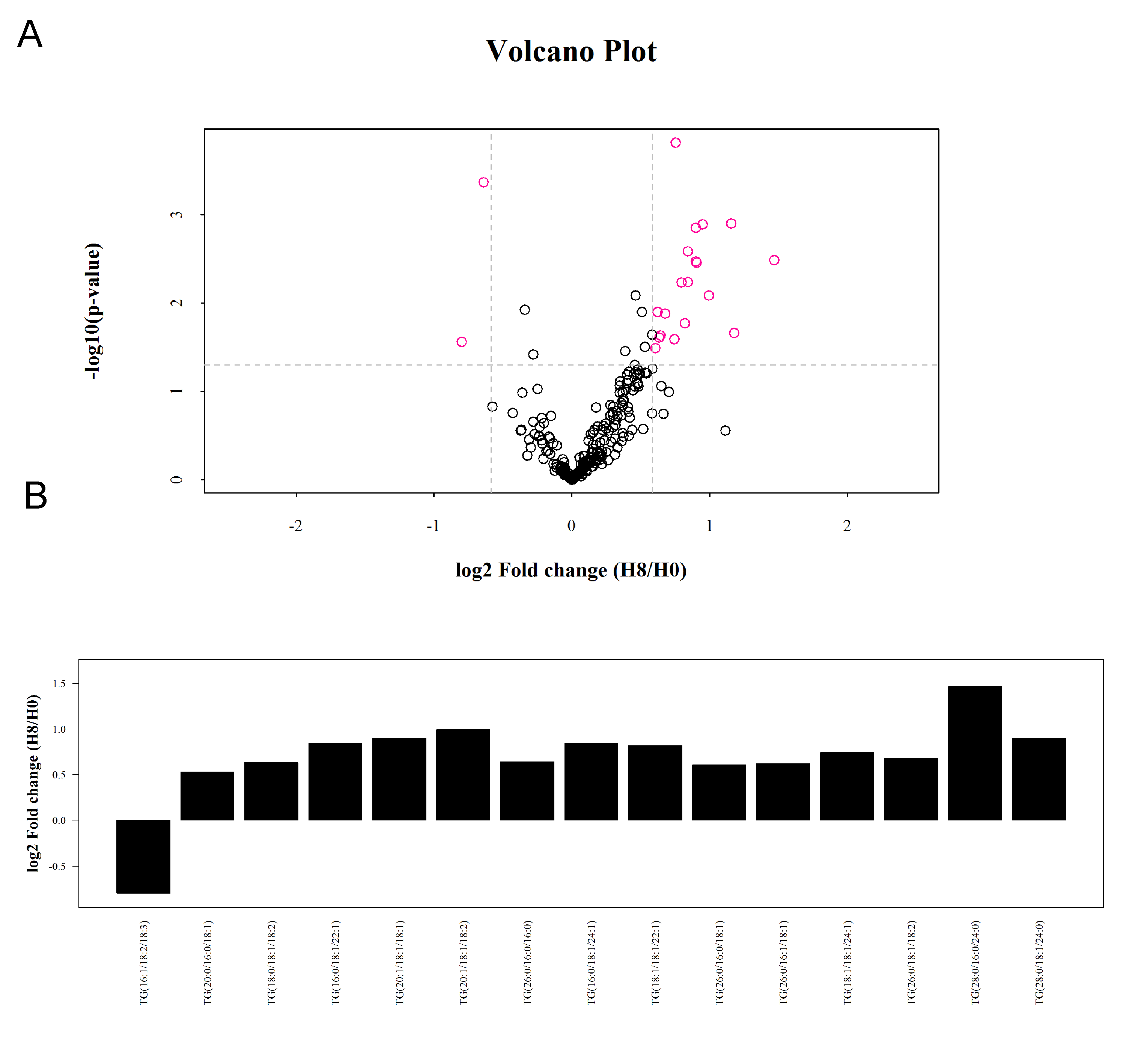


**Supplementary Figure S5.** A, volcano plot of lipid molecules divided by VIP >1 and p <0.05, red represents differently changed molecules. B, lipid molecules with significant difference between the wild-type and cyp138-knockout *Mtb* H37Rv filtered by VIP >1 and p <0.05.
