# Supplementary material for: Deletion of the Mycobacterium tuberculosis cyp138 gene leads to changes in membrane-related lipid composition and antibiotic susceptibility
Source: Front Microbiol. 2024 Mar 25;15:1301204. doi: 10.3389/fmicb.2024.1301204 (PMC10999552; doi:10.3389/fmicb.2024.1301204)
Supplement: Supplementary file 1 [file Data_Sheet_1.zip › Supplementary Figure S6.DOCX]

Supplementary Material


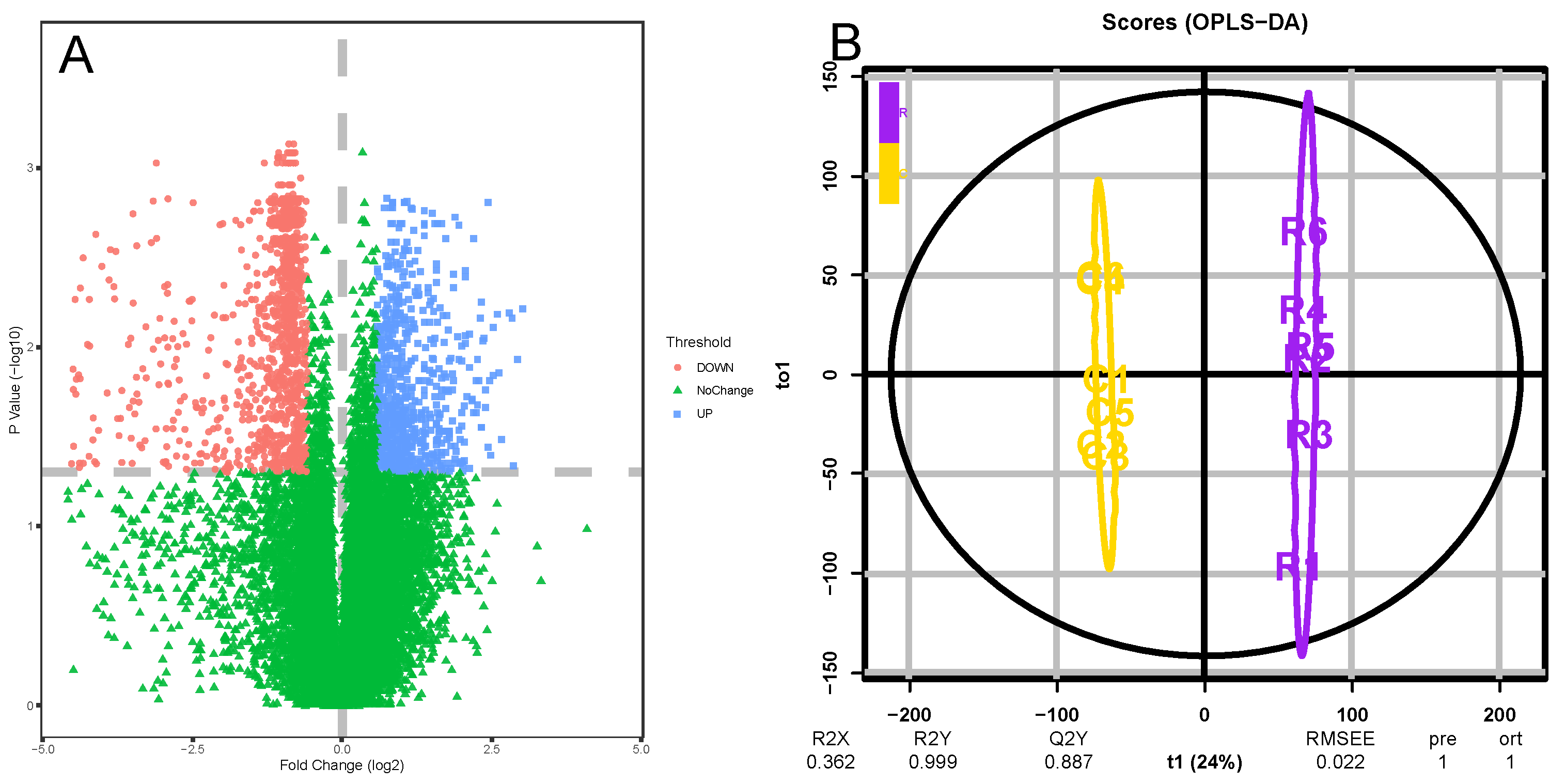


**Supplementary Figure S6.** A, volcano plot of lipid molecules annotated by MS-LAMP divided by VIP >1 and p <0.05; B, OPLS-DA results of the control and cyp138-knockout groups in *Mtb* H37Rv lipidomics analysis annotated by MS-LAMP. H0, the wild-type group; H8, the cyp138-knockout group
