# Supplementary material for: Deletion of the Mycobacterium tuberculosis cyp138 gene leads to changes in membrane-related lipid composition and antibiotic susceptibility
Source: Front Microbiol. 2024 Mar 25;15:1301204. doi: 10.3389/fmicb.2024.1301204 (PMC10999552; doi:10.3389/fmicb.2024.1301204)
Supplement: Supplementary file 1 [file Data_Sheet_1.zip › Supplementary Figure S7.DOCX]

Supplementary Material

# Results

## Observation of bacterial morphology by SEM

SEM results showed that the surface morphology of ∆138 strain was similar with that of the wild-type and ∆138-C strains (**Supplementary Figure S7**). No significant changes were observed in the morphology of the bacterial membrane and bacterial community.


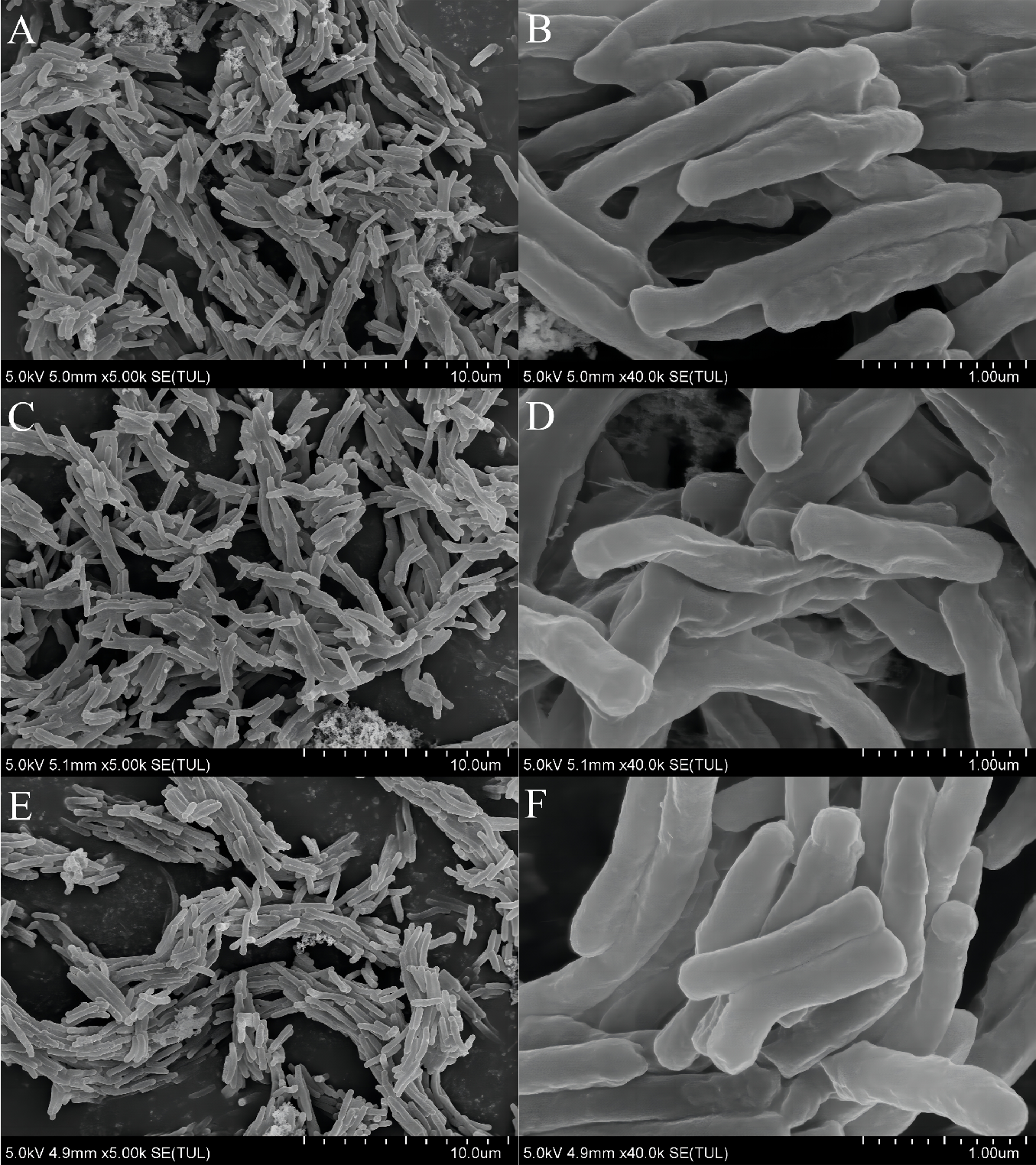


**Supplementary Figure S7.** The surface morphology of the wild-type H37Rv, ∆138 and ∆138-C strains observed by SEM.A-B, the wild-type H37Rv strain. C-D, the ∆138 strain. E-F, the ∆138-C strain. Strains were cultured in the 7H9 medium supplemented with 0.2% (vol/vol) glycerol, 0.05% (vol/vol) Tween 80, and 10% (vol/vol) OADC at 37℃ for 14 days.
