# Supplementary material for: Deletion of the Mycobacterium tuberculosis cyp138 gene leads to changes in membrane-related lipid composition and antibiotic susceptibility
Source: Front Microbiol. 2024 Mar 25;15:1301204. doi: 10.3389/fmicb.2024.1301204 (PMC10999552; doi:10.3389/fmicb.2024.1301204)
Supplement: Supplementary file 1 [file Data_Sheet_1.zip › Supplementary Table S1.DOCX]

Supplementary Material

**Supplementary Table S1.** Significantly differentially expressed proteins compared between the *cyp138*-knockout and wild-type strains filtered by p < 0.05 and fold change > 1.2.

|  | Protein names | P Value | log_2_Fold Change | Threshold |
| --- | --- | --- | --- | --- |
| I6Y778 | Probable 3-oxoacyl-[acyl-carrier protein] reductase FabG4 (3-ketoacyl-acyl carrier protein reductase) | 0.012053 | 0.284044 | UP |
| P9WH81 | 50S ribosomal protein L6 | 0.048111 | -0.27998 | DOWN |
| P96874 | Conserved protein | 0.031504 | -0.77442 | DOWN |
| O86361 | Probable acetyl-CoA acyltransferase FadA2 (3-ketoacyl-CoA thiolase) (Beta-ketothiolase) | 0.041897 | 0.281002 | UP |
| P95186 | Probable acyl-CoA dehydrogenase FadE23 | 0.011889 | 0.320114 | UP |
| P9WMF9 | DNA-binding transcriptional activator DevR/DosR | 0.047093 | -0.26658 | DOWN |
| P9WH45 | 30S ribosomal protein S19 | 0.042062 | -0.36473 | DOWN |
| I6Y0W5 | Possible acyl-CoA dehydrogenase FadE19 (MMGC) | 0.025041 | -0.28412 | DOWN |
| O53598 | Conserved protein similar to jag protein | 0.032871 | 0.276818 | UP |
| O53181 | 2-oxoglutarate oxidoreductase subunit KorB (EC 1.2.7.3) (Alpha-ketoglutarate oxidoreductase subunit beta) (KG oxidoreductase subunit beta) (KGO subunit beta) (KOR subunit beta) | 0.025786 | -0.36409 | DOWN |
| P9WPW5 | Probable succinyl-CoA:3-ketoacid coenzyme A transferase subunit A (EC 2.8.3.5) (Succinyl-CoA:3-oxoacid CoA-transferase) (OXCT A) | 0.013849 | -0.28821 | DOWN |
| P9WK55 | Lipoprotein LprA | 0.039541 | -0.3946 | DOWN |
| O53486 | Conserved protein | 0.032285 | -0.27255 | DOWN |
| P9WH69 | R2-like ligand binding oxidase (EC 1.-.-.-) (Ribonucleotide reductase R2 subunit homolog) (Ribonucleotide reductase small subunit homolog) | 0.010324 | 0.335921 | UP |
| P9WQ43 | Long-chain-fatty-acid--AMP ligase FadD26 (FAAL) (EC 6.2.1.-) (Acyl-AMP synthetase) | 0.000543 | 0.343147 | UP |
| P9WNS7 | Probable tRNA-dihydrouridine synthase (EC 1.3.1.-) | 0.032228 | 0.276446 | UP |
| P9WGI5 | RNA polymerase sigma factor SigB | 0.007482 | 0.341552 | UP |
| P9WK95 | 3-isopropylmalate dehydratase small subunit (EC 4.2.1.33) (Alpha-IPM isomerase) (IPMI) (Isopropylmalate isomerase) | 0.014914 | 0.36931 | UP |
| P9WPZ7 | Acetylornithine aminotransferase (ACOAT) (EC 2.6.1.11) | 0.030413 | -0.27876 | DOWN |
| I6Y946 | Conserved protein | 0.031974 | -0.38129 | DOWN |
| O06813 | Putative OXPP cycle protein OpcA | 0.019258 | 0.285602 | UP |
| P9WQF5 | 3-isopropylmalate dehydratase large subunit (EC 4.2.1.33) (Alpha-IPM isomerase) (IPMI) (Isopropylmalate isomerase) | 0.007792 | 0.320206 | UP |
| P9WF11 | Bifunctional NAD(P)H-hydrate repair enzyme Nnr (Nicotinamide nucleotide repair protein) [Includes: ADP-dependent (S)-NAD(P)H-hydrate dehydratase (EC 4.2.1.136) (ADP-dependent NAD(P)HX dehydratase); NAD(P)H-hydrate epimerase (EC 5.1.99.6) (NAD(P)HX epimerase)] | 0.02242 | 0.284519 | UP |
| P9WGS9 | Decaprenylphosphoryl-2-keto-beta-D-erythro-pentose reductase (EC 1.1.1.333) (Decaprenyl-phospho-2'-keto-D-arabinose reductase) (Decaprenylphospho-beta-D-erythro-pentofuranosid-2-ulose 2-reductase) (Decaprenylphosphoryl-beta-D-ribofuranose 2'-epimerase subunit DprE2) (Decaprenyl-phosphoribose 2'-epimerase subunit 2) (NAD-dependent decaprenylphosphoryl-D-2-keto-erythropentose reductase) | 0.014103 | -0.38105 | DOWN |
| P9WIR3 | Putative glyoxylase CFP32 (27 kDa antigen Cfp30B) | 0.017656 | 0.264767 | UP |
| L7N675 | PPE family protein PPE18 | 0.005881 | -0.29565 | DOWN |
| P9WME1 | Uncharacterized HTH-type transcriptional regulator Rv0196 | 0.022051 | -0.28819 | DOWN |
| O06614 | Probable PhiRv1 phage protein | 0.001736 | 0.269765 | UP |
| P9WKA1 | Signal peptidase I (SPase I) (EC 3.4.21.89) (Leader peptidase I) | 0.011247 | -0.90421 | DOWN |
| O06582 | 2-methylcitrate dehydratase (2-MC dehydratase) (EC 4.2.1.79) (Aconitate hydratase) (ACN) (Aconitase) (EC 4.2.1.3) | 0.00063 | 1.468034 | UP |
| P9WPW3 | Probable succinyl-CoA:3-ketoacid coenzyme A transferase subunit B (EC 2.8.3.5) (OXCT B) (Succinyl-CoA:3-oxoacid CoA-transferase) | 0.006007 | -0.27557 | DOWN |
| P71947 | Possible PhiRv2 prophage protein | 0.029593 | 0.339906 | UP |
| P96821 | Probable transcriptional regulatory protein (Possibly TetR-family) | 0.047423 | -0.57842 | DOWN |
| P95151 | Conserved protein | 0.002461 | 0.455252 | UP |
| P9WPM3 | Putative cytochrome P450 138 (EC 1.14.-.-) | 1.24E-06 | -2.70039 | DOWN |
| I6Y9Q3 | 2-methylcitrate synthase (2-MCS) (MCS) (EC 2.3.3.5) (Citrate synthase) (EC 2.3.3.16) | 5.74E-05 | 0.692575 | UP |
| I6Y9H2 | Possible oxidase regulatory-related protein | 0.048288 | -1.18369 | DOWN |
| P96284 | Putative inactive phenolphthiocerol synthesis polyketide synthase type I Pks15 | 0.00022 | 0.304001 | UP |
| P95105 | FMN_red domain-containing protein | 0.005736 | 0.376368 | UP |
| P9WJF3 | Cell wall synthesis protein CwsA (Cell wall synthesis and cell shape protein A) | 0.048289 | -0.86978 | DOWN |
| P9WGJ5 | Cell division protein SepF | 0.034114 | -0.27229 | DOWN |
| P9WJZ5 | Uncharacterized protein Rv2003c | 0.030189 | -0.38321 | DOWN |
| O06404 | Possible conserved membrane protein | 0.020709 | -0.9208 | DOWN |
| O06581 | HTH-type transcriptional regulator PrpR (Propionate regulator) (PRPR) | 0.005961 | 0.648593 | UP |
| P9WNC5 | Dihydroneopterin aldolase (DHNA) (EC 4.1.2.25) (7,8-dihydroneopterin 2'-epimerase) (7,8-dihydroneopterin aldolase) (7,8-dihydroneopterin epimerase) (EC 5.1.99.8) (7,8-dihydroneopterin hydroxylase) (EC 1.13.11.81) (Dihydroneopterin epimerase) (Dihydroneopterin hydroxylase) | 0.046579 | -0.6673 | DOWN |
| P9WJI1 | Probable ferredoxin/ferredoxin--NADP reductase (FNR) (EC 1.18.1.2) | 0.007471 | 0.340334 | UP |
| I6Y2E2 | Probable monooxygenase | 0.00405 | 0.465289 | UP |
| O05574 | Conserved serine rich protein | 0.001696 | -0.31228 | DOWN |
| O53673 | Heat shock protein Hsp (Heat-stress-induced ribosome-binding protein A) | 0.038962 | 0.51108 | UP |
| O33362 | Possible membrane acyltransferase | 0.020729 | 0.511771 | UP |
| P9WGY3 | Putative 4-hydroxy-4-methyl-2-oxoglutarate aldolase (HMG aldolase) (EC 4.1.3.17) (Oxaloacetate decarboxylase) (OAA decarboxylase) (EC 4.1.1.112) (Regulator of ribonuclease activity homolog) (RraA-like protein) | 0.049512 | -0.26484 | DOWN |
| O53530 | Uncharacterized protein | 0.023526 | 0.454414 | UP |
| P9WJ91 | Probable antitoxin MazE4 | 0.009628 | -0.31865 | DOWN |
| I6X8R5 | Heme-binding protein Rv0203 | 0.014911 | 0.299809 | UP |
| P9WJR1 | Molybdopterin synthase catalytic subunit 2 (EC 2.8.1.12) (MPT synthase subunit 2 2) (Molybdenum cofactor biosynthesis protein E 2) (Molybdopterin-converting factor large subunit 2) (Molybdopterin-converting factor subunit 2 2) | 0.040793 | 0.523249 | UP |
| O05453 | ESAT-6-like protein EsxD | 0.038715 | -0.68892 | DOWN |
| I6XWF9 | Uncharacterized protein | 0.042206 | 0.334816 | UP |
| P9WL03 | Uncharacterized protein Rv0313 | 0.031316 | 0.766306 | UP |
| P9WFD1 | Universal stress protein Rv2026c (USP Rv2026c) | 0.03389 | 0.506182 | UP |
| P9WJY9 | Homoserine O-acetyltransferase (HAT) (EC 2.3.1.31) (Homoserine transacetylase) (HTA) | 0.029123 | 0.263768 | UP |
| P9WKW3 | Uncharacterized protein Rv0441c | 0.003046 | 0.289212 | UP |
| I6YFL7 | Rubredoxin | 0.036222 | 0.33529 | UP |
| I6X9X6 | Methyltranfer_dom domain-containing protein | 0.019493 | -0.6486 | DOWN |
| P9WLP3 | Uncharacterized protein Rv1995 | 0.009462 | 0.363314 | UP |
| I6YDH3 | Conserved protein | 0.011109 | -1.05317 | DOWN |
| P9WLY7 | Uncharacterized protein Rv1405c | 0.016382 | 0.322263 | UP |
| L7N6A2 | Possible transcriptional regulatory protein (Possibly TetR-family) | 0.010365 | 0.331053 | UP |
| P9WIN5 | Phthiocerol/phthiodiolone dimycocerosyl transferase (EC 2.3.1.282) (Acyltransferase PapA5) (Phthiocerol/phthiodiolone O-acyltransferase) (Polyketide synthase-associated protein A5) | 0.04537 | -0.43389 | DOWN |
| P9WJU1 | Acyltrehalose exporter MmpL10 | 0.011653 | 0.456251 | UP |
| P9WLV3 | Uncharacterized protein Rv1525 | 0.006426 | 0.419903 | UP |
| P9WIH1 | PE family immunomodulator PE15 | 0.008407 | 0.485773 | UP |
| P9WH01 | Ribonuclease HII (RNase HII) (EC 3.1.26.4) | 0.010884 | 0.277018 | UP |
| P96356 | Uncharacterized protein | 0.011367 | 0.319403 | UP |
| P9WGG7 | ECF RNA polymerase sigma factor SigE (ECF sigma factor SigE) (Alternative RNA polymerase sigma factor SigE) (RNA polymerase sigma-E factor) (Sigma-E factor) | 0.014448 | 0.458205 | UP |
| P9WQD3 | Holo-[acyl-carrier-protein] synthase (Holo-ACP synthase) (EC 2.7.8.7) (4'-phosphopantetheinyl transferase AcpS) | 0.028887 | -0.65226 | DOWN |
| P96375 | Conserved protein | 0.013395 | 0.295579 | UP |
| O53413 | Uncharacterized protein | 0.021123 | 0.682853 | UP |
| P9WPP3 | Methyl-branched lipid omega-hydroxylase (EC 1.14.15.14) (Cholest-4-en-3-one C26-monooxygenase) (Cholest-4-en-3-one C26-monooxygenase [(25R)-3-oxocholest-4-en-26-oate forming]) (Cholesterol C26-monooxygenase) (Cholesterol C26-monooxygenase [(25R)-3beta-hydroxycholest-5-en-26-oate forming]) (Cytochrome P450 124) (Steroid C26-monooxygenase) (EC 1.14.15.28) (Steroid C27-monooxygenase) | 0.000382 | 0.281876 | UP |
| I6XA42 | GCN5-related N-acetyltransferase | 0.044093 | 0.271222 | UP |
| P9WN29 | Bifunctional uridylyltransferase/uridylyl-removing enzyme (UTase/UR) (Bifunctional [protein-PII] modification enzyme) (Bifunctional nitrogen sensor protein) [Includes: [Protein-PII] uridylyltransferase (PII uridylyltransferase) (UTase) (EC 2.7.7.59); [Protein-PII]-UMP uridylyl-removing enzyme (UR) (EC 3.1.4.-)] | 0.020704 | 0.385547 | UP |
| P9WIW5 | NADH-quinone oxidoreductase subunit M (EC 7.1.1.-) (NADH dehydrogenase I subunit M) (NDH-1 subunit M) | 0.044885 | 0.26358 | UP |
| P9WLB7 | Uncharacterized protein Rv2313c | 0.025834 | 0.284653 | UP |
| O50430 | Low molecular weight T-cell antigen TB8.4 | 0.035704 | -0.30213 | DOWN |
| O06805 | ANTAR domain-containing protein | 0.025447 | 0.571532 | UP |
| Q6MWY3 | Probable MoaD-MoaE fusion protein MoaX | 0.037935 | 0.296023 | UP |
| P9WK43 | Putative lipoprotein LprH | 0.002698 | -0.32951 | DOWN |
| P9WIG7 | PE family immunomodulator PE35 | 0.009857 | -0.29644 | DOWN |
| P9WNB9 | Endonuclease 8 1 (DNA glycosylase/AP lyase Nei 1) (EC 3.2.2.-) (DNA-(apurinic or apyrimidinic site) lyase Nei 1) (EC 4.2.99.18) (Endonuclease VIII 1) | 0.017244 | 0.429064 | UP |
| P0DN33 | Uncharacterized protein Rv0609B | 0.029359 | -0.76329 | DOWN |
| P9WLF7 | Uncharacterized protein Rv2271 | 0.042741 | -2.18651 | DOWN |
| I6XG38 | HNHc domain-containing protein | 0.023543 | 0.285187 | UP |
| O07747 | Resuscitation-promoting factor RpfC (EC 3.-.-.-) | 0.039628 | 0.393384 | UP |
| P9WMH7 | Transcriptional regulator ClgR | 0.010679 | 0.611482 | UP |
| P9WL35 | Uncharacterized protein Rv2886c | 0.04352 | 0.411839 | UP |
| P95021 | Uncharacterized protein | 0.010139 | 0.28413 | UP |
| O07745 | Uncharacterized protein | 0.012781 | 0.272732 | UP |
| P95278 | Possible monooxygenase | 0.003404 | 0.322192 | UP |
| P9WNF7 | Putative FAD-containing monooxygenase MymA (EC 1.14.13.-) | 0.021152 | 0.354385 | UP |
| O50440 | Diacyltrehalose acyltransferase Chp2 (EC 2.3.1.-) | 0.046355 | 0.326806 | UP |
| O07423 | Possible lipoprotein LprO | 0.025205 | 0.351117 | UP |
| P9WKQ1 | Sphingomyelinase (SMase) (EC 3.1.4.12) | 0.035816 | 0.348903 | UP |
| P9WIF7 | Uncharacterized PE-PGRS family protein PE_PGRS24 | 0.040657 | 0.299042 | UP |
| P9WG99 | Sec-independent protein translocase protein TatB | 0.00151 | 0.287735 | UP |
| P9WNE9 | NADPH oxidoreductase (EC 1.-.-.-) (Stearoyl-CoA 9-desaturase electron transfer partner) | 0.03647 | 0.385636 | UP |
| O05894 | Rubredoxin | 0.027514 | 0.383131 | UP |
| P9WKC5 | Probable diacyglycerol O-acyltransferase tgs3 (TGS3) (EC 2.3.1.20) (Probable triacylglycerol synthase tgs3) | 0.012331 | 0.340468 | UP |
| I6YA50 | Possible conserved transmembrane alanine rich protein | 0.016346 | 0.480619 | UP |
| P9WJ97 | Isoniazid-induced protein IniB | 0.002112 | 0.537112 | UP |
| P9WQ65 | Probable ammonia channel (Ammonia transporter) | 0.012163 | 0.54755 | UP |
