# Supplementary material for: Deletion of the Mycobacterium tuberculosis cyp138 gene leads to changes in membrane-related lipid composition and antibiotic susceptibility
Source: Front Microbiol. 2024 Mar 25;15:1301204. doi: 10.3389/fmicb.2024.1301204 (PMC10999552; doi:10.3389/fmicb.2024.1301204)
Supplement: Supplementary file 1 [file Data_Sheet_1.zip › Supplementary Table S2.DOCX]

Supplementary Material

**Supplementary Table S2.** Parameters of the PCA and OPLS-DA analysis of lipidomics comparison of the wild-type and *cyp138*-knockout strains.

| Type | A | R^2^X  （cum） | R^2^Y  （cum） | Q^2^  （cum） |
| --- | --- | --- | --- | --- |
| PCA | 2 | 0.861 |  | 0.735 |
| OPLS-DA | 1+1+0 | 0.86 | 0.885 | 0.742 |
