# Supplementary material for: Deletion of the Mycobacterium tuberculosis cyp138 gene leads to changes in membrane-related lipid composition and antibiotic susceptibility
Source: Front Microbiol. 2024 Mar 25;15:1301204. doi: 10.3389/fmicb.2024.1301204 (PMC10999552; doi:10.3389/fmicb.2024.1301204)
Supplement: Supplementary file 1 [file Data_Sheet_1.zip › Supplementary Table S4.DOCX]

Supplementary Material

**Supplementary Table S4.** PRM targeted proteome results of peptides in the wild-type, *cyp138*-knockout, and *cyp138*-complement strains.

| Peptides | Uniprot ID | Proteins | Fold change (∆138/ wild-type) | t test (∆138/ wild-type) | Fold change (∆138 /∆138-C) | t test (∆138-C/∆138-C) |
| --- | --- | --- | --- | --- | --- | --- |
| AMSSLGSSLGSSGLGGGVAANLGR | L7N675 | PPE18 | 0.3107 | 0.0014 | 0.2679 | 0.0090 |
| ALPLTSLTSAAER | L7N675 | PPE18 | 0.1263 | 0.0037 | 0.2361 | 0.0098 |
| GPGQMLGGLPVGQMGAR | L7N675 | PPE18 | 0.2287 | 0.0014 | 0.2674 | 0.0019 |
| ALEGFTR | I6Y778 | FabG4 | 1.2944 | 0.0389 | 1.4904 | 0.0161 |
| VAIVTGAAR | I6Y778 | FabG4 | 1.2505 | 0.0228 | 1.5244 | 0.0209 |
| DGAHVVAIDVESAAENLAETASK | I6Y778 | FabG4 | 1.9416 | 0.0117 | 1.6652 | 0.0266 |
| VVAEVLR | P9WQF5 | LeuC | 1.1498 | 0.0457 | 1.4684 | 0.0219 |
| SFVAAPGR | P9WJ97 | IniB | 2.8577 | 0.0006 | 2.2530 | 0.0008 |
| IIEAYAK | I6Y2E2 | Probable monooxygenase | 1.6758 | 0.0111 | 1.9363 | 0.0102 |
